# Cytotoxic Activity of Sicilian Red- and White-Grape Seed Oils on Human Liver and Colorectal Cancer Cells

**DOI:** 10.3390/molecules31101567

**Published:** 2026-05-08

**Authors:** Daniela Ganci, Giulia Abruscato, Roberto Chiarelli, Manuela Mauro, Vincenzo Arizza, Mirella Vazzana, Claudio Luparello

**Affiliations:** 1 Dipartimento di Scienze e Tecnologie Biologiche Chimiche e Farmaceutiche (STEBICEF), Università di Palermo, 90128 Palermo, Italy; daniela.ganci01@unipa.it (D.G.); giulia.abruscato@unipa.it (G.A.); roberto.chiarelli@unipa.it (R.C.); manuela.mauro01@unipa.it (M.M.); vincenzo.arizza@unipa.it (V.A.); mirella.vazzana@unipa.it (M.V.); 2 NBFC—National Biodiversity Future Center, 90133 Palermo, Italy

**Keywords:** Beclin-1, p62, LC3, MLKL, caspase-1, gasdermin-D, HMGB1, hsp60, hsp90, reactive oxygen species

## Abstract

Seed oils from Sicilian white (WGSO) and red grapes (RGSO) were examined for their possible cytotoxic effect on HepG2 liver and CaCo-2 colorectal cancer cells, the latter also induced to intestinal differentiation. Half maximal inhibitory dilution (ID_50_) values were obtained from viability assays, excluding RGSO-treated HepG2 and differentiated CaCo-2 cells exposed to both oils, which were unresponsive. Cell morphology and cycle status, reactive oxygen species (ROS) production, and the levels of cytoprotection, regulated cell death (RCD), and autophagy markers were evaluated. No occurrence of canonical apoptosis was proven in any experimental condition. In HepG2 cells, WGSO ID_50_ primarily triggered autophagy collapse, as evidenced by modulation of Beclin-1, p62 and LC3 markers, initiating a cascade of metabolic disturbances that led to oxidative stress reaction and mild inflammatory signaling. In CaCo-2 cells, WGSO ID_50_ mainly elicited a strong ROS-mediated cell injury without major alterations in autophagy, with transient activation but incomplete execution of pyroptotic and necroptotic effectors (gasdermin-D, pMLKL and HMGB1). In the same cells, RGSO ID_50_ induced a weaker metabolic perturbation with transient activation of multiple RCD pathways and concomitant autophagy inhibition. Research findings revealed distinct damage-inducing properties linked to oils’ chemical profiles, underscoring their prospective utilization as beneficial bioactive supplements.

## 1. Introduction

Approximately 30% of the total grapes used in wine production end up as a substantial amount of waste. Presently, the transformation of winemaking by-products into valuable resources is a key circular economy strategy, employing recycling methods within a restorative paradigm that enhances resource efficacy, mitigates environmental degradation and fosters economic development [1]. Among these discarded materials are grape seeds, valued for their abundant oil content offering nutraceutical and health benefits due to their richness in phenolic compounds, polyunsaturated fatty acids, vitamins and phytosterols. The qualitative and quantitative attributes of these components are modulated by the specific grape cultivar, prevailing environmental factors and maturation stage of the seeds [2,3]. Research increasingly indicates that the phytochemical components of grape seed oils manifest a broad spectrum of activities, including anti-inflammatory, antioxidant, hypoglycemic, cardioprotective, antimicrobial, and anticancer effects [4,5].

To date, the extant scientific literature provides sparse evidence concerning the in vitro and in vivo cytotoxic effects of grape seed oils on tumor cells. A study by Shaban et al. [6] highlighted the significant anticancer potential of the saponifiable components derived from red- (RGSO) and white-grape seed oils (WGSO) against MCF-7 breast cancer cells and Ehrlich ascites carcinoma mouse models. This was attributed to their ability to promote apoptosis and induce oxidative stress, and their predicted competitive inhibition on the cancer migration-associated enzymes metalloproteinase-9 and cathepsin B. An enhanced anticarcinogenic and pro-apoptotic activity was recently documented for a nanoemulsified formulation of grape seed oil when evaluated against the Ehrlich ascites tumor model [7]. Moreover, Al-Ashmawy et al. [8] and Abu-Serie et al. [9] demonstrated that grape seed oils induce cell cycle arrest and activate the apoptotic machinery in cultured cancer cells of different derivation, as well as in hepatocellular carcinoma-bearing mice.

We have previously reported that WGSO and RGSO sourced from specific Sicilian cultivars grown in the provinces of Palermo, Trapani and Agrigento (Western Sicily, Italy) efficiently stimulate glucose absorption and glycogenesis in vitro, via their modulation of the protein expression of GLUT2 and GLUT4 glucose transporters and elements of the related signaling pathways [10]. Prompted by increasing scientific evidence indicating that several bioactive components within Mediterranean food industry by-products engage molecular mechanisms targeting hallmarks of cancer [11], here we explored the potential cytotoxic effects of the oils extracted from the Sicilian white and red grapes against cancer cells in vitro. Two cell lines modeling digestive system tumors, i.e., HepG2 (hepatic cancer) and CaCo-2 (colorectal cancer), were utilized in the experimental set. Furthermore, Caco-2 cells spontaneously differentiated into intestinal epithelial cells (diff-CaCo-2) [12] were also included in the analysis as normaloid counterparts. Thus, using a combination of flow cytometric and protein blotting techniques, once the oils’ half maximal inhibitory dilutions (ID_50_) were determined, a comparative evaluation of their impact on cellular redox status, induction of regulated cell death (RCD) and cytoprotective mechanisms, and impairment of the autophagic process was performed.

## 2. Results

### 2.1. Effects of WGSO and RGSO on Cell Viability

Pure-WGSO and -RGSO samples were weighted, establishing that a volume of 1 mL corresponded to approximately 1.27 mg and 1.03 mg, respectively. An initial 1:20 stock solution of the oils, corresponding to 63.5 mg/mL for WGSO and 51.5 mg/mL for RGSO, was prepared in fetal bovine serum, after which various dilutions of these preparations in a complete culture medium were applied to HepG2, CaCo-2 or diff-CaCo-2 cells for 24 h. Following the incubation, their morphological appearance was preliminarily examined using a light microscope. Visible alterations, like cell shrinkage, rounding and detachment, were evident in WGSO-treated HepG2 and CaCo-2 cell cultures, starting from 1:100 to 1:80 dilutions, and also in RGSO-treated CaCo-2 cell cultures at all tested dilutions, starting from 1:320. In contrast, neither the HepG2 cell cultures treated with RGSO nor the diff-CaCo-2 cell cultures exposed to either oil type displayed any detectable modification. Panels of phase-contrast micrographs showing the cell cultures across the different experimental conditions are available in Figure S1.

**Figure 1:**
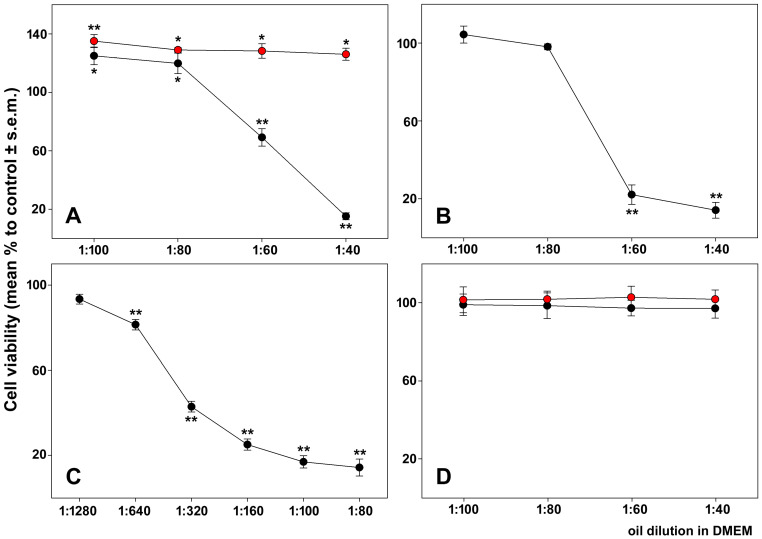
Dilution-response effect of the oils on the viability of cells after 24 h of exposure. (**A**) HepG2 cells treated with WGSO (black circles) and RGSO (red circles); (**B**) CaCo-2 cells treated with WGSO; (**C**) CaCo-2 cells treated with RGSO; (**D**) diff-CaCo-2 cells treated with WGSO (black circles) and RGSO (red circles). The error bars correspond to the standard error of the mean (s.e.m.) of biological triplicates. One-way ANOVA followed by Holm–Sidak post hoc test was used. * *p* < 0.05; ** *p* < 0.001 compared to control. Normality test passed.

Results from the viability test using 3-(4,5-dimethylthiazol-2-yl)-2,5 diphenyl tetrazolium bromide (MTT) were in agreement with the microscopical findings. As shown in Figure 1A, HepG2 cells exposed for 24 h to WGSO at dilutions from 1:40 to 1:100 exhibited a small rise in cell viability at the lower dilutions, followed by a dose-dependent reduction. In contrast, RGSO had no effect at the tested dilutions, but instead led to limited yet stable enhancement of cell viability. Conversely, the CaCo-2 cell line displayed dose–response curves when treated with both oils (Figure 1B,C), exhibiting significantly greater susceptibility to the action of RGSO in highly diluted conditions after a 24 h exposure. It is noteworthy that the MTT test confirmed the absence of cytotoxic effects of WGSO and RGSO on diff-CaCo-2 cells (Figure 1D).

In all subsequent biological investigations, which sought to characterize the molecular basis of the cytotoxic activity of both oils on CaCo-2 cells and of WGSO on HepG2 cells, the mean half maximal inhibitory dilutions (ID_50_) were consistently employed. These were estimated from the analysis of the obtained dilution-response curves and corresponded to 1:52 (about 24.4 mg/mL) for WGSO-treated HepG2 cells, 1:64 (about 19.8 mg/mL) for WGSO-treated CaCo-2 cells, and 1:554 (about 1.9 mg/mL) for RGSO-treated CaCo-2 cells.

### 2.2. Effects of WGSO and RGSO ID_50_ on Cell Morphology, Cell Cycle Status and the Levels of RCD Markers

**Figure 2:**
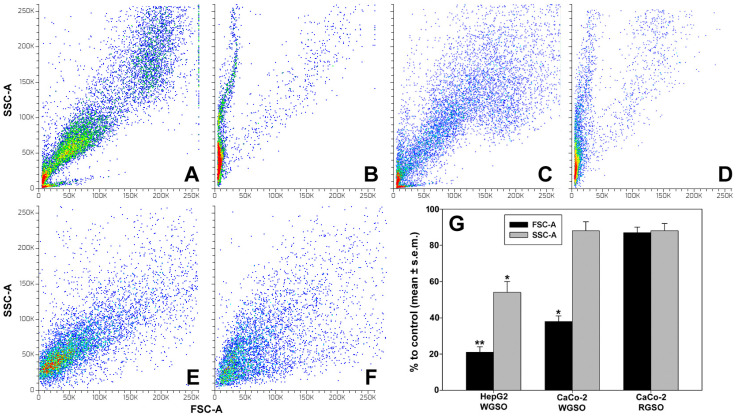
Representative density plots for FSC-A and SSC-A in HepG2 cells (**A**,**B**) and CaCo-2 cells (**C**–**F**) cultured in control conditions (**A**,**C**,**E**) or exposed to the ID_50_ of WGSO (**B**,**D**) or RGSO (**F**) for 24 h. (**G**) Bar graph showing the percentage of FSC-A and SSC-A values compared to control conditions normalized to 1. Values are expressed as mean % ± standard error of the mean (s.e.m). Each bar is representative of biological triplicates. One-way ANOVA followed by Holm–Sidak comparison procedure was used. Normality test passed. * *p* < 0.05, ** *p* < 0.001 compared to control.

Given the observed decrease in cell viability following treatment with the oils, we submitted control and exposed cells to a set of experiments aimed to evaluate the impact of WGSO and RGSO ID_50_ on cell size and complexity, measured via forward-scatter (FSC-A) and side-scatter (SSC-A) flow cytometry analysis, cell cycle status, and eventual onset of RCD processes. Consistent with the microscopical observations, as shown in Figure 2B,G, HepG2 cells exposed to WGSO exhibited a substantial reduction in average FSC-A (about 21% vs. control) and a lesser but significant decline in average SSC-A (about 54% vs. control), indicative of pronounced cell stress characterized by cell shrinkage, organelle disruption and potential fragmentation. In contrast, WGSO treatment of CaCo-2 cells resulted exclusively in a slightly diminished average FSC-A (about 38% vs. control; Figure 2D,G), indicative of cellular retraction, rounding and subsequent detachment, without affecting organelle integrity. No alterations in FSC-A and SSC-A metrics were observed in RGSO-treated CaCo-2 cells (Figure 2F,G).

**Figure 3:**
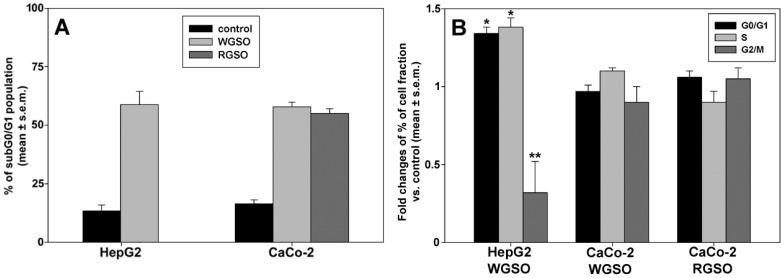
Effect of WGSO and RGSO ID_50_ administration for 24 h on HepG2 and CaCo-2 cell cycle status. (**A**) Bar graph showing the percentage of cells in the subG_0_/G_1_ fraction in control and treated conditions. Values are expressed as mean % ± standard error of the mean (s.e.m). (**B**) Bar graph showing the treatment-induced changes in the cell cycle phase distribution for the surviving cell population. Values are expressed as mean fold change ± standard error of the mean (s.e.m.) compared to the controls. Each bar is representative of biological triplicates. One-way ANOVA followed by Holm–Sidak comparison procedure was used. Normality test passed. * *p* < 0.05, ** *p* < 0.001 compared to control.

Therefore, we checked whether the oils could interfere with the cell cycle phase distribution and alter the percentage of cells in the subG_0_/G_1_ fraction. As shown in Figure 3, exposure of cells to the ID_50_ of the oils for 24 h induced an approximate mean increment in the subG_0_/G_1_ fraction of 45% for WSGO-treated HepG2 cells, 41% for WGSO-treated CaCo-2 cells, and 39% for RSGO-treated CaCo-2 cells vs. controls (Figure 3A). Analysis of DNA profiles of the fraction of surviving cells (Figure 3B) indicated that only WGSO-treated HepG2 cells exhibited approximately 34% and 38% increases in G_0_/G_1_ and S cell fractions, respectively, alongside an approximate 32% reduction in the G_2_/M cell fraction, when compared to the control. Conversely, neither WGSO nor RGSO elicited statistically significant changes in the cell cycle progression of surviving CaCo-2 cells.

**Figure 4:**
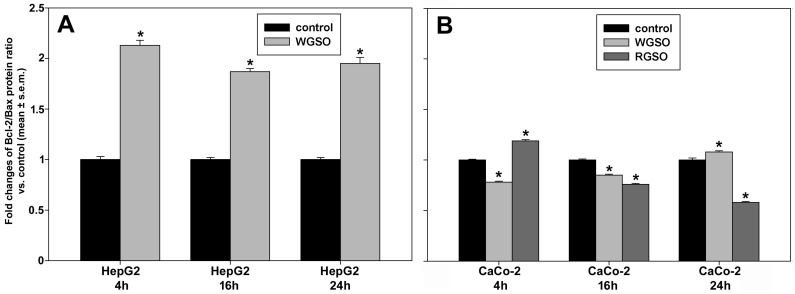
Effect of WGSO or RGSO ID_50_ administration for 4, 16 and 24 h on the Bcl-2/Bax protein ratio in HepG2 cells (**A**) and CaCo-2 cells (**B**). The original immunoblots are shown in Figure S2A–F. Each bar is representative of technical triplicates. Values are expressed as mean fold change ± standard error of the mean (s.e.m.) compared to the controls. One-way ANOVA followed by Holm–Sidak comparison procedure was used. Normality test passed. * *p* < 0.001 compared to control.

Subsequently, we investigated if the observed rise in subG_0_/G_1_ hypodiploid cell percentage was attributable to an apoptosis-promoting effect of the oils. To this purpose, initially, the Bcl-2/Bax protein ratio was evaluated via Western blot analysis in both the control and oil-treated samples. Indeed, it is recognized that the regulation of the intrinsic apoptotic pathway depends primarily on the stoichiometric ratio of Bcl-2 to Bax rather than on their individual abundance [13]. As shown in Figure 4, the Bcl-2/Bax protein ratio in HepG2 cells was up-regulated by 1.13 ± 0.05, 0.87 ± 0.03 and 0.95 ± 0.06 fold vs. controls, following 4, 16 and 24 h of exposure to WGSO, respectively (Figure 4A). WGSO induced less pronounced alterations in CaCo-2 cells, characterized by a reduction in the ratio to 78 ± 0.01% and 85 ± 0.04% vs. controls after 4 and 16 h of exposure, followed by a modest but significant up-regulation of 8 ± 0.01% vs. control. Regarding CaCo-2 cell exposure to RGSO, the chronological response exhibited an opposite trend, that is, an initial up-regulation of the ratio of 19 ± 0.01% vs. control, followed by a reduction to 85 ± 0.01% at 16 h and a further drop to 58 ± 0.01% vs. controls by 24 h.

**Figure 5:**
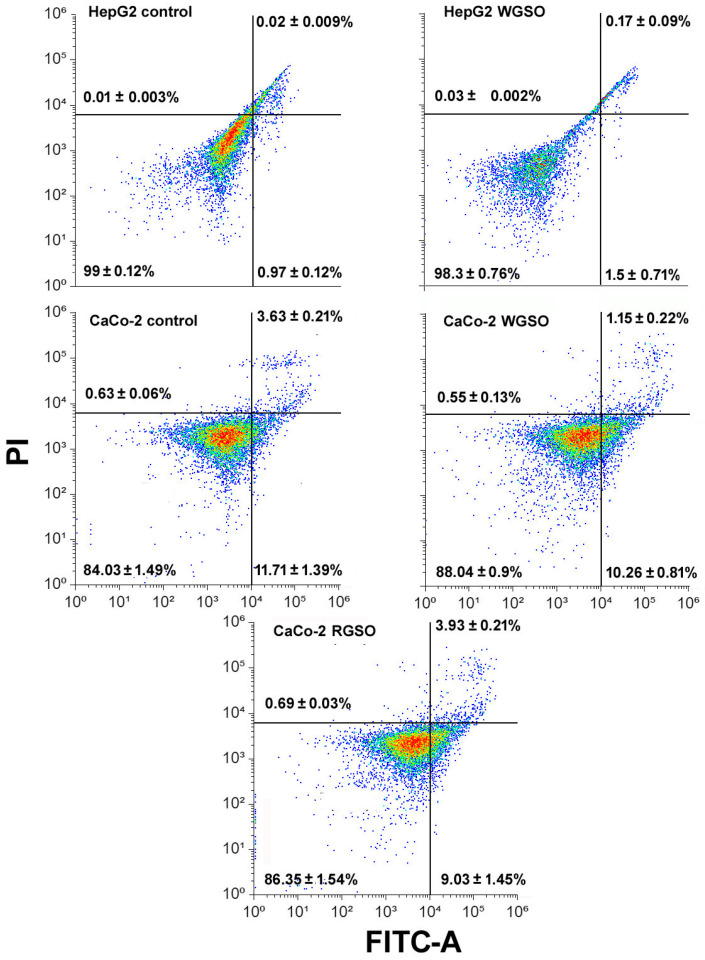
Flow cytometric assays for externalization of phosphatidylserine in HepG2 and CaCo-2 cells cultured in control conditions or exposed to the ID_50_ of the oils for 24 h. The plots show the results of representative experiments and the percentages, indicated as the mean ± standard error of the mean (s.e.m.) of biological triplicates, refer to viable annexinV-negative/PI-negative cells (bottom-left quadrant), early-apoptotic annexinV-positive/PI-negative cells (bottom-right quadrant), late-apoptotic annexinV-positive/PI-positive cells (top-right quadrant), and necrotic annexinV-negative/PI-positive cells (top-left quadrant).

We then evaluated the amount of cells positive for AnnexinV, an indicator of apoptosis-promoted phosphatidylserine translocation from the inner to the outer leaflet of the plasma membrane, after 24 h of treatment. Flow cytometry analyses involved dual staining with AnnexinV and propidium iodide (PI), a red-fluorescent DNA stain for cells displaying membrane damage associated with late apoptosis or necrosis. The data obtained showed no significant differences in the distribution of early- or late-apoptotic cell populations across all experimental groups (Figure 5).

**Figure 6:**
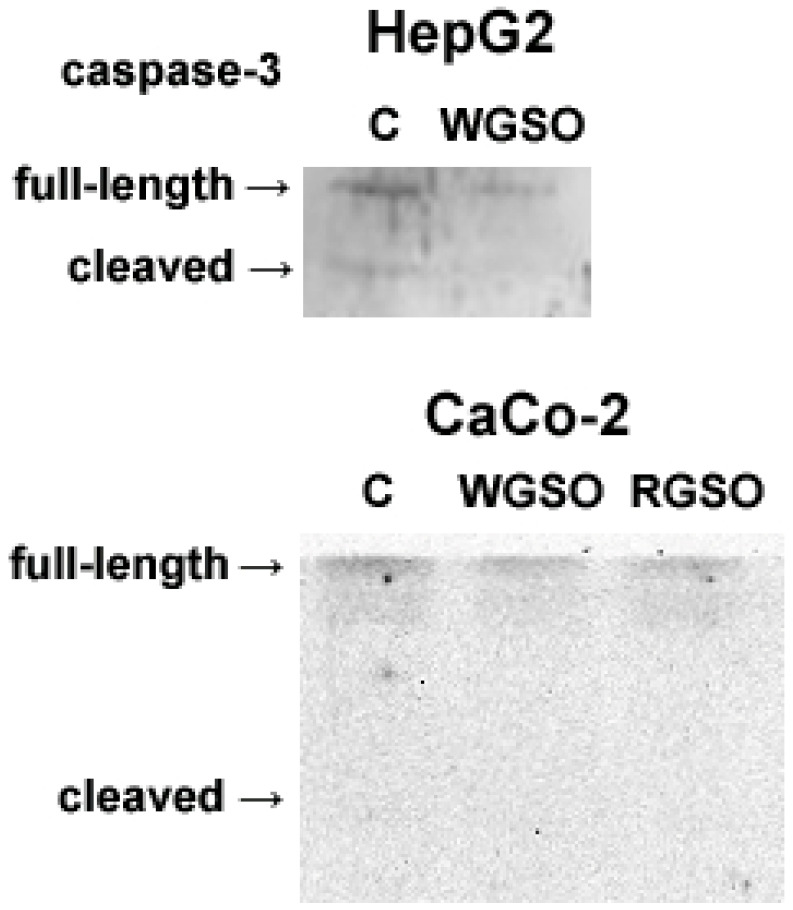
Western blot analysis for the activation of caspase-3 in control and oil-treated HepG2 and CaCo-2 cells. The complete immunoblots are shown in Figure S2G,H.

Furthermore, the potential activation of caspase-3 was assessed following the same duration of oil exposure. Consistently, as shown in Figure 6, no proteolytic activation of caspase-3 was detected following the exposure of the cells to either WGSO or RGSO for 24 h. Comparable results were observed also at 4 h and 16 h; as an example, data from Western blot analysis of CaCo-2 cells under these reduced exposure times are presented in Figure S2H.

As markers of classical apoptosis were not identified, we explored alternative RCD pathways by analyzing the intracellular levels of both cleaved caspase-1/cleaved gasdermin-D and phosphorylated mixed-lineage kinase domain-like protein (pMLKL), which are both linked to cell membrane breakdown and serve as biomarkers of pyroptosis and necroptosis, respectively [14,15]. In addition, the extracellular levels of high-mobility group box 1 protein (HMGB1), which is an indicator of necrosis/necroptosis and oxidative stress-driven autophagic cell death [16], were also evaluated.

**Figure 7:**
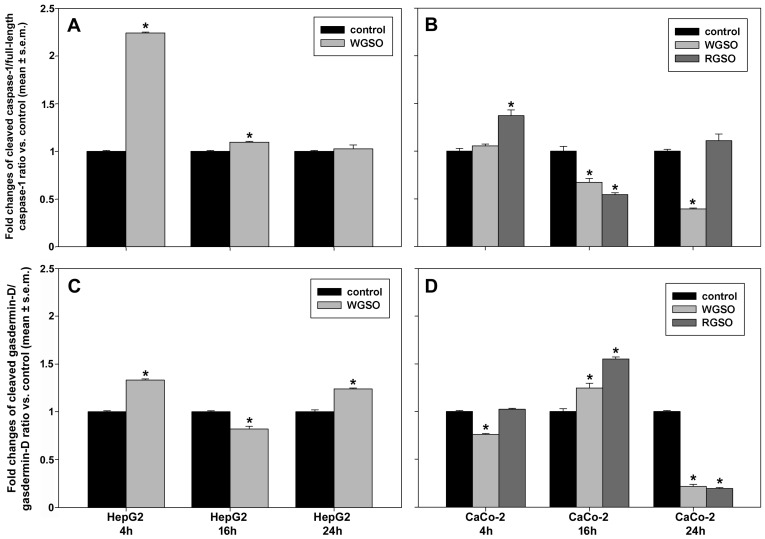
Effect of WGSO or RGSO ID_50_ administration for 4, 16 and 24 h on the cleaved caspase-1/full-length caspase-1 ratio (**A**,**B**) and the cleaved gasdermin-D/gasdermin-D ratio (**C**,**D**) in HepG2 cells (**A**,**C**) and CaCo-2 cells (**B**,**D**). The original immunoblots are shown in Figure S2I–M. Each bar is representative of technical triplicates. Values are expressed as mean fold change ± standard error of the mean (s.e.m.) compared to the controls. One-way ANOVA followed by Holm–Sidak comparison procedure was used. Normality test passed. * *p* < 0.001 compared to control.

Figure 7A,B present the data regarding the oil-induced changes in the ratios of activated (cleaved) caspase-1 to full-length caspase-1 proenzyme. In HepG2 cells (Figure 7A), WGSO prompted a marked early increase of 1.24 ± 0.01 fold which subsequently declined to 0.1 ± 0.01 fold vs. control after 16 h of exposure, reaching equivalence with the untreated sample by 24 h. In contrast, as shown in Figure 7B, WGSO treatment failed to elicit an initial response in CaCo-2 cells, although it induced a subsequent decline in the ratio to 67.4 ± 0.04% and 39.3 ± 0.01% at 16 and 24 h vs. controls, respectively. In the same cells, RGSO treatment initially stimulated a 37.1 ± 0.06% increase in the ratio compared to the control, followed by a subsequent decrease to 54.4 ± 0.02% of the control after 16 h of incubation, ultimately reaching equivalence with the untreated specimen at 24 h. The ratio of cleaved gasdermin-D to total gasdermin-D, a common substrate for caspase-1 characterized by an aminoterminal pore-forming domain [17], was also assessed under the same conditions (Figure 7C,D). As shown in Figure 7C, in WGSO-treated HepG2 cells the ratio underwent an initial up-regulation of 33.2 ± 0.01% vs. control, then declined to 81.7 ± 0.03% of the control at 16 h, finally reaching a 23.9 ± 0.01% increase over control after 24 h of exposure. WGSO induced an inverse phenomenon in CaCo-2 cells, characterized by an initial down-regulation of the ratio to 75.9 ± 0.01% of the control, followed by an increase of 24.6 ± 0.05% at 16 h, before subsequently declining to 21.5 ± 0.02% by 24 h. Upon treatment with RGSO, Caco-2 cells exhibited a marked ratio increase of 55 ± 0.02% vs. control at 16 h, followed, also in this case, by a sharp decrement to 19.3 ± 0.01% of the control at 24 h, whereas no changes were observed at the initial time point (Figure 7D).

**Figure 8:**
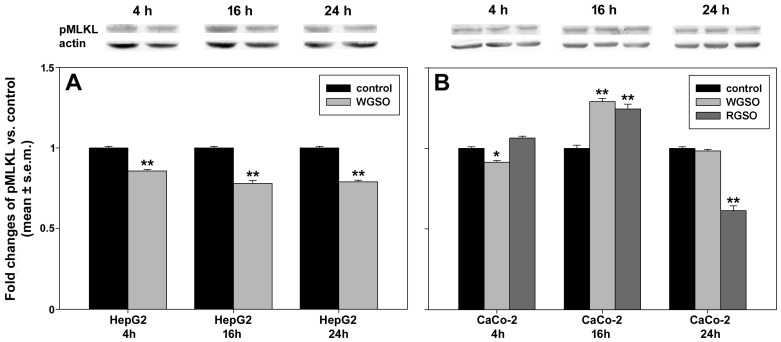
Effect of WGSO or RGSO ID_50_ administration for 4, 16 and 24 h on pMLKL protein levels in HepG2 (**A**) and CaCo-2 cells (**B**), determined by Western blot. The complete immunoblots are shown in Figure S2N,O. Each bar is representative of technical triplicates. Values are expressed as mean fold change ± standard error of the mean (s.e.m.) compared to the controls. One-way ANOVA followed by Holm–Sidak comparison procedure was used. Normality test passed. * *p* < 0.05, ** *p* < 0.001 compared to control.

Changes in intracellular pMLKL protein accumulation following oil administration to HepG2 and CaCo-2 cells are depicted in Figure 8. In particular, HepG2 cells treated with WGSO (Figure 8A) showed slightly but consistently lower pMLKL levels vs. controls across all time points (85.5 ± 0.01%, 77.8 ± 0.01% and 78.9 ± 0.01% at 4, 16 and 24 h, respectively). In contrast, CaCo-2 cells exposed to WGSO exhibited a 29 ± 0.02% increase vs. control at 16 h, with no statistically significant changes at earlier and later times. A comparable pattern occurred in RGSO-treated CaCo-2 cells, which showed an up-regulation of 24.5 ± 0.02% vs. control at 16 h followed by a significant decrease to 61.3 ± 0.04% of control at 24 h (Figure 8B).

**Figure 9:**
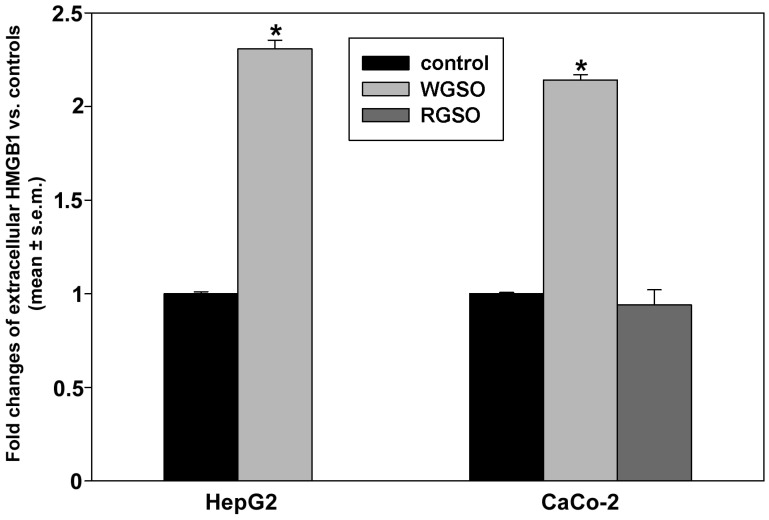
Effect of WGSO or RGSO ID_50_ on the amount of HMGB1 protein released in the media of HepG2 and CaCo-2 cells after 24 h, determined by dot blot. The original immunoblot is shown in Figure S2P. Each bar is representative of technical triplicates. Values are expressed as mean fold change ± standard error of the mean (s.e.m.) compared to the controls. One-way ANOVA followed by Holm–Sidak comparison procedure was used. Normality test passed. * *p* < 0.001 compared to control.

Assessment of extracellular HMGB1 through dot blot analysis (Figure 9) indicated that 24 h incubation with WGSO resulted in 1.3 ± 0.03 and 1.14 ± 0.04 fold increases vs. controls in the media of HepG2 and CaCo-2 cell cultures, respectively. Conversely, the abundance of immunoreactive HMGB1 remained unaltered in the medium of CaCo-2 cell cultures after 24 h of RGSO exposure.

### 2.3. Effects of WGSO and RGSO ID_50_ on hsp60 and hsp90 Protein Levels

**Figure 10:**
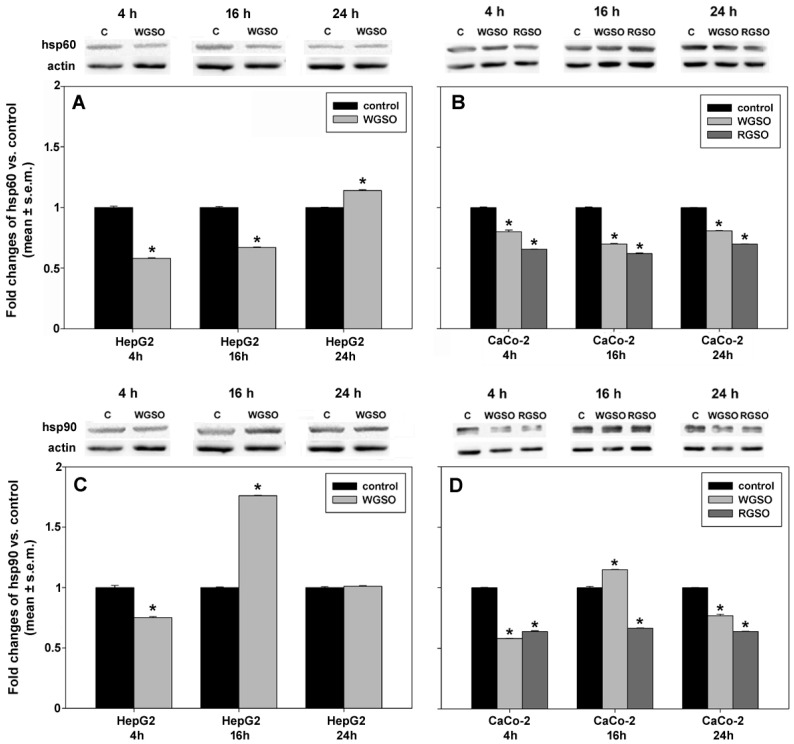
Effect of WGSO or RGSO ID_50_ administration for 4, 16 and 24 h on hsp60 (**A**,**B**) and hsp90 (**C**,**D**) protein levels in HepG2 (**A**,**C**) and CaCo-2 cells (**B**,**D**), determined by Western blot. The complete immunoblots are shown in Figure S2Q–S. Each bar is representative of technical triplicates. Values are expressed as mean fold change ± standard error of the mean (s.e.m.) compared to the controls. One-way ANOVA followed by Holm–Sidak comparison procedure was used. Normality test passed. * *p* < 0.001 compared to control.

We quantified the levels of mitochondrial hsp60 and cytosolic hsp90 proteins in control conditions and after 4, 16 or 24 h of exposure of the cells to the oils. These proteins are established indicators of cytoprotection, being essential components of the protein folding apparatus [18,19,20,21]. As shown in Figure 10A, the treatment of HepG2 cells with WGSO led to an initial decline of hsp60 levels (58 ± 0.06% of the control after 4 h), followed by a subsequent rise to 67 ± 0.04% and 114 ± 0.07% of the controls at 16 and 24 h, respectively. The time-dependent response concerning hsp90 levels showed an inverted-U-shaped pattern, characterized by an initial decrease to 75 ± 0.06% of control at 4 h, followed by a significant up-regulation to 176 ± 0.05% of control by 16 h and a decrement to values comparable to those of the control after 24 h (Figure 10C). Exposure of CaCo-2 cells to the oils resulted in consistent decreases in hsp60 levels (Figure 10B), accounting for 80 ± 0.01%, 70 ± 0.03% and 81 ± 0.01% of control values for WGSO and 66 ± 0.03%, 62 ± 0.05% and 70 ± 0.01% for RGSO at 4, 16 and 24 h post-oil administration. Similarly, as shown in Figure 10D, RGSO-treated cells displayed a sustained reduction in hsp90 levels over the entire duration of the assay (64 ± 0.05%, 67 ± 0.02% and 64 ± 0.01% of the controls after 4, 16 and 24 h, respectively). In contrast, analogously to HepG2 cells, WGSO-exposed CaCo-2 cells also exhibited an inverted-U-shaped pattern in hsp90 levels, characterized by an initial down-regulation to 58 ± 0.03% of the control at 4 h, followed by an increase to 115 ± 0.02% of the control at 16 h and a subsequent decline to 77 ± 0.01% of the control at 24 h.

### 2.4. Effects of WGSO and RGSO ID_50_ on the Production of Reactive Oxygen Species (ROS)

**Figure 11:**
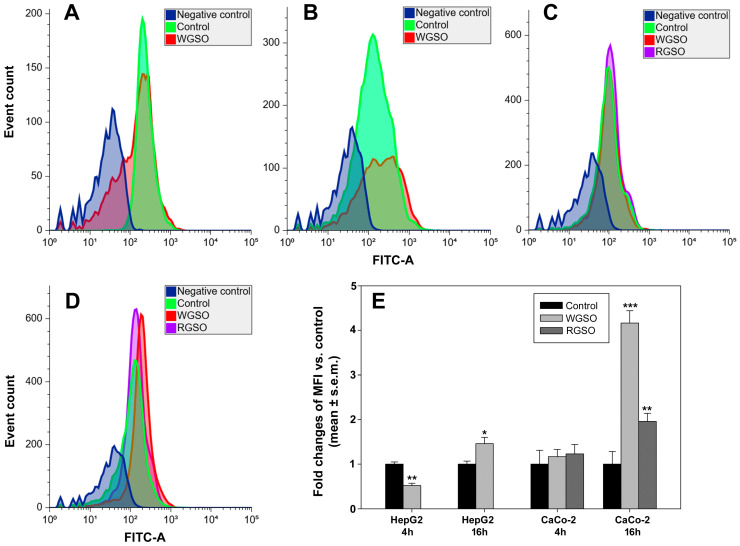
Representative flow cytometric profiles for ROS production in HepG2 cells (**A**,**B**) and CaCo-2 cells (**C**,**D**) cultured in control conditions or exposed to the ID_50_ of WGSO or RGSO for 4 (**A**,**C**) or 16 h (**B**,**D**). (**E**) Bar graph showing the MFI of ROS indicator, obtained from triplicate experiments. Cumulative data from flow cytometry experiments were analyzed for the MFI of each condition; data normalization was then achieved by dividing the MFI of the treated cell samples by the MFI of the reference controls. Each bar is representative of biological triplicates. Values are expressed as mean fold change ± standard error of the mean (s.e.m.) compared to the controls. One-way ANOVA followed by Holm–Sidak comparison procedure was used. Normality test passed. * *p* < 0.01, ** *p* < 0.05, *** *p* < 0.001 compared to control.

The production of ROS by HepG2 and CaCo-2 cells was examined by flow cytometry at early (4 h) and intermediate (16 h) stages of exposure to the oils. The data presented in Figure 11 reveal a modest biphasic alteration of ROS production by WGSO-treated HepG2 cells, that is, an initial down-regulation of 0.52 ± 0.05 fold vs. control at 4 h, followed by a subsequent increase of 0.46 ± 0.14 fold vs. control after 16 h of incubation (Figure 11A,B,E). When ROS production by treated CaCo-2 cells was examined, a statistically significant up-regulation of 0.96 ± 0.18 fold and, more prominently, 3.16 ± 0.28 fold, was observed for RGSO- and WGSO-treated cells after 16 h of exposure, respectively. Conversely, no changes were detected at the earlier time point (Figure 11C–E).

### 2.5. Effects of WGSO and RGSO ID_50_ on the Levels of Autophagic Markers

To ascertain the potential impact of the oils on the autophagic process in HepG2 and CaCo-2 cells, we quantified both the accumulation of acidic vesicular organelles (AVOs) and the intracellular levels of the protein markers LC3 (specifically, the soluble LC3-I and the membrane-bound LC3-II forms), Beclin-1 and p62 (a.k.a. SQSTM1, i.e., sequestosome 1) at early (4 h) and intermediate (16 h) post-treatment time points.

**Figure 12:**
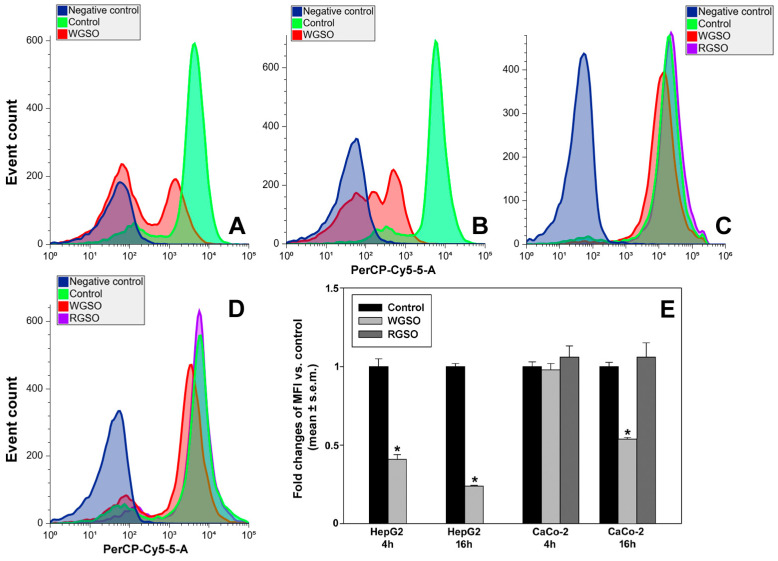
Representative flow cytometric profiles for AVOs’ accumulation in HepG2 cells (**A**,**B**) and CaCo-2 cells (**C**,**D**) cultured in control conditions or exposed to the ID_50_ of WGSO or RGSO for 4 (**A**,**C**) or 16 h (**B**,**D**). (**E**) Bar graph showing the mean fluorescence index (MFI) of acridine orange stain, obtained from triplicate experiments. Cumulative data from flow cytometry experiments were analyzed for the MFI of each condition; data normalization was then achieved by dividing the MFI of the treated cell samples by the MFI of the reference controls. Each bar is representative of biological triplicates. Values are expressed as mean fold change ± standard error of the mean (s.e.m.) compared to the controls. One-way ANOVA followed by Holm–Sidak comparison procedure was used. Normality test passed. * *p* < 0.001 compared to control.

As indicated by the flow cytometry data in Figure 12, the exposure of both HepG2 and CaCo-2 cells to WGSO led to a reduction in the amount of AVOs. In particular, treated HepG2 cells showed a sharp decline in acridine orange fluorescent signal already at the earliest time point, reaching 41 ± 0.03% vs. control and further decreasing to 24 ± 0.003% vs. control after 16 h of incubation (Figure 12A,B,E). Also, the fraction of acridine orange-negative cells increased from 17.6 ± 4.4% and 10.8 ± 3% in the control samples to 55.6 ± 3.4% and 51.7 ± 2.8% in the treated samples after 4 and 16 h of culture, respectively. Differently, the AVO-dependent mean fluorescence index (MFI) in CaCo-2 cells incubated with WGSO was initially comparable to the control. Nevertheless, a prolonged 16 h-treatment resulted in a decrease down to 54 ± 0.01% vs. control, suggesting a more gradual and milder action of the oil on AVO down-regulation in this cytotype. Across the time intervals examined, in these cells the already low percentage of acridine orange-negative cells did not exhibit any statistically significant modification upon treatment. Furthermore, no measurable variations in both AVOs’ and acridine orange-negative cells’ amounts were observed in CaCo-2 cells incubated with RGSO compared to controls during analogous incubation periods (Figure 12C–E).

**Figure 13:**
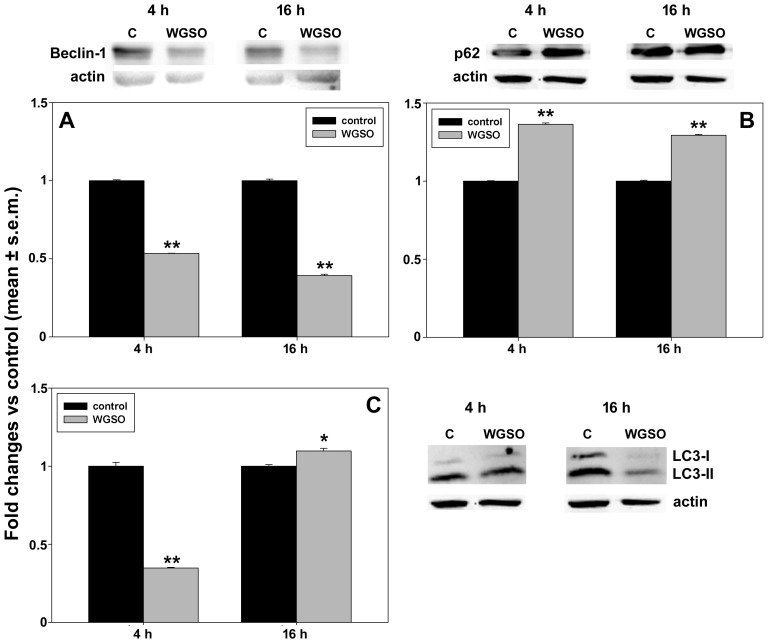
Effect of WGSO ID_50_ administration for 4 and 16 h on Beclin-1 (**A**) and p62 (**B**) protein levels and LC3-II/LC3-I protein ratio (**C**) in HepG2 cells, determined by Western blot. The complete immunoblots are shown in Figure S2T,U. Each bar is representative of technical triplicates. Values are expressed as mean fold change ± standard error of the mean (s.e.m.) compared to the controls. One-way ANOVA followed by Holm–Sidak comparison procedure was used. Normality test passed. * *p* < 0.05, ** *p* < 0.001 compared to control.

**Figure 14:**
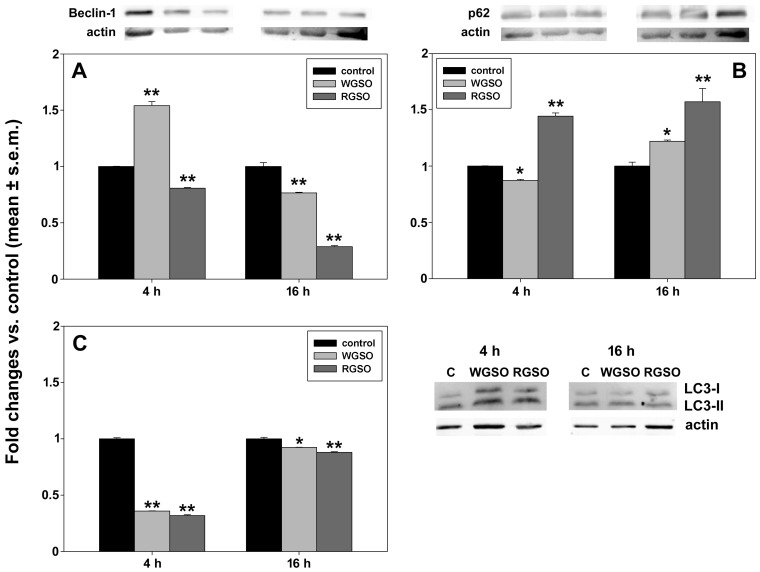
Effect of WGSO or RGSO ID_50_ administration for 4 and 16 h on Beclin-1 (**A**) and p62 (**B**) protein levels and LC3-II/LC3-I protein ratio (**C**) in CaCo-2 cells, determined by Western blot. The complete immunoblots are shown in Figure S2V–X. Each bar is representative of technical triplicates. Values are expressed as mean fold change ± standard error of the mean (s.e.m.) compared to the controls. One-way ANOVA followed by Holm–Sidak comparison procedure was used. Normality test passed. * *p* < 0.05, ** *p* < 0.001 compared to control.

Western blot analyses of the autophagy-related protein levels are presented in Figure 13 (for HepG2 cells) and Figure 14 (for CaCo-2 cells). Treatment of HepG2 cells with WGSO resulted in a downward trend for Beclin-1, with its levels reaching 53.4 ± 0.01% and 39.2 ± 0.09% of control values at 4 and 16 h from the start of the experiment, respectively (Figure 13A). Conversely, p62 levels increased by 36.2 ± 0.09% and 29.3 ± 0.05% vs. controls at the same time points (Figure 13B). We also examined the LC3-II/LC3-I ratio, which decreased to 34.8 ± 0.04% of the control after 4 h of treatment, but then showed a minor yet statistically significant increase of 9.7 ± 0.02% vs. control after 16 h of incubation (Figure 13C).

On the other hand, differently from HepG2 cells, WGSO treatment of CaCo-2 cells elicited a biphasic response for Beclin-1, initially increasing by 54 ± 0.04% of the control and later declining to 76.6 ± 0.06% of the control after 16 h of exposure (Figure 14A). For p62, an initial minor decrease to 87.3 ± 0.09% of the control was observed, followed by an up-regulation of 22 ± 0.01% vs. control after 16 h of treatment (Figure 14B). A decline in the LC3-II/LC3-I ratio to 36 ± 0.02% of the control was observed at 4 h after the administration, with a subsequent elevation to 92.5 ± 0.08% of the control by the 16 h time point (Figure 14C). In these cells, RGSO caused a down-regulation of Beclin-1 to 80.7 ± 0.05% of the control after 4 h of exposure and 28.9 ± 0.1% of the control following 16 h of treatment (Figure 14A). Also in this context, a significant up-regulation of p62 was detected, showing increases of 44.4 ± 0.03% at 4 h and 57.1 ± 0.19% at 16 h relative to controls (Figure 14B). This was accompanied by a concomitant down-regulation of the LC3-II/LC3-I ratio with a trend that attenuated over the experimental duration, quantified as 31.9 ± 0.07% and 88 ± 0.06% of the control values at 4 h and 16 h, respectively (Figure 14C).

## 3. Discussion

The production of bioactive extracts for nutraceutical and biomedical applications has emerged as a focal point among the numerous strategies suggested for the proficient valorization of grape seed oil residues [22,23,24]. The present research sought to assess the cytotoxic efficacy of RGSO and WGSO, derived from Sicilian winery by-products, on two hepatic and colorectal cancer cell lines, alongside an analysis of the associated molecular targets. The dilutions selected for this study were derived from the ID_50_ values obtained via MTT assays, ensuring a robust correlation between dose and biological effect. While these concentrations might exceed systemic plasma levels, they are representative of the local concentrations achievable in the intestinal lumen following the ingestion of grape seed oil-enriched functional foods. The CaCo-2 model, in particular, mimics the direct exposure of the intestinal mucosa to dietary lipids. For the hepatic model, these doses allowed for the identification of specific signaling pathways, providing a mechanistic basis that can be further refined in future in vivo dose-escalation studies. Notably, ID_50_ was established for WGSO-treated HepG2 and WGSO- and RGSO-treated CaCo-2 cells; conversely, HepG2 cells showed no reduction in viability at any RGSO concentration tested, preventing further biological investigation in this model. Of particular importance are the results of the viability tests on differentiated intestinal CaCo-2 cells, upon which neither WGSO nor RGSO demonstrated toxicity, thereby enhancing the prospective utility of the preparations for future applications.

The overall data collected demonstrate that the two Sicilian grape seed oils exert distinct and cytotype-dependent effects and suggest that they primarily elicit stress-related cellular reactions, regulating autophagy, redox homeostasis, and inflammatory signaling, instead of classical RCD mechanisms.

Furthermore, our findings reveal a downward trend in the magnitude of oil-dependent biochemical perturbations, with maximal potency observed in HepG2 cells exposed to WGSO, followed by a decrease across CaCo-2 cells treated with WGSO and RGSO, respectively, as detailed in the following section:

Strong WGSO effect on HepG2 cells: A decline in FSC-A and SSC-A levels was found in exposed HepG2 cells, indicative of a reduction in both cell volume and intracellular complexity and consistent with metabolic imbalance and compromised organelle homeostasis [25]. The pronounced suppression of autophagic activity was the most significant mechanistic response. In particular, the marked reduction in acridine orange-positive AVOs, the down-regulation of Beclin-1 expression and the diminished conversion of LC3-II, combined with the accumulation of the autophagy substrate p62, confirmed that WGSO interferes with autophagy initiation and execution, thereby reducing autophagosome formation and impairing the degradation of cellular components [26,27]. Since autophagy is fundamentally involved in the preservation of cellular homeostasis through the elimination of compromised organelles and misfolded proteins, the severe impairment of the autophagic process observed may contribute to metabolic stress and cell cycle arrest. In hepatic cancer cells, where energy balance is sustained through intensive lipid metabolism and autophagic turnover [28], defective autophagic clearance has been found to lead to substantial biological outcomes, such as accumulation of damaged mitochondria and protein aggregates [29]. Thus, autophagic dysregulation is plausibly implicated in the progressive cellular stress observed during WGSO treatment of HepG2 cells.

Consistent with the impairment of autophagic turnover, HepG2 cells exposed to WGSO displayed dynamic shifts in the levels of ROS with a biphasic response involving an initial reduction followed by a later increase. This suggests that the oil’s components exerted an initial antioxidant effect that is subsequently overwhelmed by oxidative stress, possibly triggered by the accumulation of dysfunctional mitochondria resulting from inefficient mitophagy and/or impaired cellular detoxification mechanisms. This hypothesis is supported by the concurrent increase in stress-related hsp90 protein, which is typically up-regulated during proteotoxic and oxidative stress [30,31]. In addition, the early reduction in hsp60 levels followed by a gradual recovery and overexpression at 24 h confirms the initial cellular stress response and implies a later compensatory induction of mitochondrial chaperone systems, presumably to restore proteostasis under stress conditions. Collectively, these phenomena indicate the establishment of metabolic stress resulting from compromised cellular homeostasis.

The metabolic perturbations were accompanied by substantial shifts in the cell cycle profile, characterized by the WGSO-induced enrichment of cells in the G_0_/G_1_ and S phases and a parallel depletion of the G_2_/M fraction. This suggests that the autophagy inhibition-related stress induced by the oil interferes with transition into mitosis, leading to cell cycle arrest and suppression of proliferation. The increase in the subG_0_/G_1_ fraction induced by WGSO treatment in the absence of correlation with classical apoptotic markers supports the occurrence of DNA damage associated with cell death processes linked to autophagy dysregulation and redox perturbation [32].

In agreement with this perspective, the WGSO reaction in HepG2 cells additionally exhibited the transient activation of inflammatory signaling pathways. Caspase-1 and gasdermin-D are central components of the canonical inflammasome pathway. Following activation, caspase-1 catalyzes the cleavage of gasdermin-D, which releases its N-terminal fragment, thereby promoting the formation of transmembrane pores that switch on pyroptosis, a lytic form of inflammatory RCD [33]. Necroptosis constitutes a regulated form of inflammatory necrosis driven by the Receptor-Interacting Protein 3 kinase (RIP3)-induced phosphorylation of MLKL protein, which promotes its translocation to the plasmalemma and the consequent generation of membrane pores via interactions with phosphatidylinositol bisphosphate [34]. The protein HMGB1, physiologically situated in the nucleus and bound to chromatin, is released into the extracellular space through membrane pores generated during cell stress or death, where it functions as an “alarmin” and a damage-associated molecular pattern (DAMP), thereby driving significant pro-inflammatory signaling [35]. The release of HMGB1 may be facilitated by activated MLKL, and, in turn, extracellular HMGB1 may activate caspase-1 [36,37]. In treated HepG2 cells, early activation of caspase-1 and increased extracellular HMGB1 release point toward the induction of DAMP signalization, yet the limited cleavage of gasdermin-D suggests that a comprehensive pyroptotic response is not fully realized. Therefore, such evidence might represent a sub-threshold activation of the inflammasome that fails to culminate in full inflammatory lysis [38].

In summary, the cumulative data suggest that WGSO primarily induces the collapse of the autophagic process; this critical event initiates a cascade of metabolic disturbances that result in a cytotoxic stress response accompanied by mild inflammatory signaling.

Moderate WGSO effect on CaCo-2 cells: In contrast to the pronounced alterations observed in HepG2 cells, WGSO-treated Caco-2 cells displayed a more moderate biochemical dysregulation. Although a modest reduction in FSC-A suggests a marginal influence of the oil on cell dimensions, the cell cycle distribution within the surviving treated population remained largely unaffected. Of note, WGSO stimulated a robust increase in ROS levels, suggesting that the response of colorectal cancer cells is primarily and heavily influenced by oxidative stress pathways. On the other hand, despite the significant elevation in ROS production, especially at intermediate time points, the autophagy markers indicated a more dynamic regulation of the autophagic process than that observed in HepG2 cells. Evidence of early Beclin-1 accumulation paired with a decline in p62 levels suggests a transient activation of autophagy, which might constitute an attempt to activate an adaptive mechanism enabling cells’ survival under metabolic pressure [39]. This response eventually dissipates, presumably due to sustained oxidative stress, leading to a transition from cytoprotection to non-regulated ROS-driven cell damage. Even the temporary induction of hsp90 may be interpreted as a component of the cellular safeguarding strategy, whereas the persistent reduction in hsp60 implies a likely enduring deficit in mitochondrial protein-folding efficiency.

Also in this cell line, WGSO-induced cytotoxicity appears to diverge from canonical pyroptotic or necroptotic pathways. In fact, a substantial ROS increase and transient elevations in cleaved gasdermin-D and phosphorylated MLKL levels at 16 h occurred without caspase-1 activation, excluding classical inflammasome-mediated pyroptosis. At 24 h, reduced gasdermin-D cleavage and caspase-1 activity suggest a failure of regulated lytic death, potentially caused by oxidative damage to key regulatory proteins and loss of cellular homeostasis. Consistently, only a modest increase in extracellular HMGB1 was detected, supporting the occurrence of limited membrane permeabilization and, by analogy with WGSO-treated HepG2 cells, stress-induced DAMP release rather than extensive lytic breakdown.

In summary, the cumulative data support the hypothesis that WGSO triggers ROS-mediated, non-canonical lytic cell injury without major alterations in autophagy. This response is likely associated with transient activation but incomplete execution of pyroptotic and necroptotic effectors, culminating in oxidative-stress-driven cell death rather than a defined RCD pathway.

Weak RGSO effect on CaCo-2 cells: The Bcl-2/Bax ratio of RGSO-treated CaCo-2 cells showed an early survival response followed by a pro-death shift. However, the lack of executioner caspase activity suggests incomplete or restrained mitochondrial signaling. This “apoptotic priming” without execution may reflect a sublethal stress condition in which cells approach but do not reach the threshold for irreversible commitment to apoptosis [40]. A transient modulation of markers of pyroptosis and necroptosis was also observed. The early activation of caspase-1 and the cleavage of gasdermin D, along with the increased pMLKL levels at 16 h, suggest the triggering of inflammasome and necroptotic pathways. Nonetheless, the return to the baseline or the down-regulation of these markers by 24 h, together with the lack of extracellular HMGB1, indicates that neither pyroptosis nor necroptosis is finalized. The concurrent, yet incomplete, induction of apoptotic, pyroptotic, and necroptotic pathways suggests a convergent signaling state in which multiple RCD programs are engaged but fail to drive terminal cell death, possibly due to competitive constraints or insufficient signal magnitude.

Similar to the autophagic suppression observed in WGSO-treated HepG2 cells, CaCo-2 cells showed a comparable inhibition of this process when exposed to RGSO, as indicated by the down-regulation of Beclin-1, the increment of p62, and the reduction in the LC3-II/LC3-I ratio. This is further supported by the absence of changes in AVO levels. Although compromised autophagic activity may exacerbate the accumulation of misfolded proteins and damaged organelles, the lack of those morphological or cell cycle-related consequences observed in WGSO-treated HepG2 cells suggests that the extent of autophagy inhibition induced by RGSO in CaCo-2 cells does not reach the threshold to cause major cellular dysfunction.

Indeed, both hsp60 and hsp90 levels were significantly decreased at all examined time points, evidencing a disturbance in proteostasis. Since these chaperones are fundamental for protein folding and maintain the stability of key signaling molecules [18,19,20,21], their down-regulation likely contributes to the destabilization of survival pathways and further sensitizes cells to stress. Notably, oxidative stress seemed to have a negligible impact, given that only a modest increase in ROS levels was observed, suggesting that RGSO does not primarily act as a strong pro-oxidant on this cell line.

In summary, the cumulative data support the hypothesis that RGSO induces a generally weak metabolic perturbation with a state of multifactorial stress characterized by inhibition of autophagy and disruption of proteostasis alongside the transient activation of multiple RCD pathways.

While a detailed study of the molecular pathways underlying the oils’ activity requires further analyses, as referenced in Table 1, the current scientific literature suggests that specific polyphenolic constituents within the oils could be correlated with the biological responses identified in HepG2, CaCo-2 and, more broadly, hepatic and colorectal cancer cells.

Further evidence of comparable biological efficacy emerges from the analysis of other cytotypes. In fact, hydroxytyrosol has been shown to substantially reduce hsp60 levels in lipopolysaccharide-stimulated macrophages [53]. Ferulic acid demonstrated the ability to prevent apoptosis in PC-12 pheochromocytoma cells, inhibit autophagy in cervical cancer cells, decrease hsp90 in melanoma cells and trigger pyroptosis in lung cancer cells by regulating caspase-1 and gasdermin D through the ROS/c-Jun N-terminal kinase (JNK)/Bax mitochondrial pathway [54,55,56,57]. Oleocanthal was found to promote extensive permeabilization of the lysosome membrane in several cancer cell lines, facilitating abrupt necrotic death in vitro as well as increased survival rates in vivo for pancreatic neuroendocrine tumor models [58]. Also, the suppressive effect of rutin on autophagy, evidenced by a JNK signaling-dependent reduction in Beclin-1 levels, was further demonstrated in glioblastoma cells [59] as well as in the hepatic tissues of rats treated with valproic acid [60]. Moreover, kaempferol has been proven to induce the extracellular release of HMGB1 by RMCCA-1 cholangiocarcinoma cells [61], and to down-regulate hsp90 levels in a mouse model of age-related diminished ovarian reserve [62].

It is worth mentioning that the potential involvement of uncharacterized constituents of Sicilian WGSO and RGSO, such as phytosterols, carotenoids and volatile substances [4], in the reported biological activity must also be taken into account, as well as the influence of the minimal amount of unesterified lipids, or the possible synergistic interactions among the extant compounds.

## 4. Materials and Methods

### 4.1. Cell Cultures and Grape Seed Oils

HepG2 liver tumor cells (RRID: CVCL_0027) and CaCo-2 colorectal cancer cells (RRID: CVCL_0025), primarily obtained from ATCC (Manassas, VA, USA) and sourced from the laboratory’s internal stocks, were routinely cultured in a high-glucose Dulbecco’s Modified Eagle’s medium (DMEM; D6429, Sigma, St. Louis, MO, USA) enriched with 10% heat-inactivated fetal bovine serum (FBS, F4135, Sigma) and antibiotics (100 U/mL penicillin and 100 mg/mL streptomycin; Capricorn Scientific GmbH, Ebsdorfergrund, Germany) within a humidified environment at 37 °C under a 5% CO_2_ atmosphere. Spontaneously differentiated CaCo-2 cells were obtained as described by Cicio et al. [63] and maintained in culture under the previously mentioned conditions. WGSO and RGSO, derived from the by-products of either Sangiovese red grapes or a white grape blend consisting of 26% Cataratto, 54% Insolia, and 20% Grillo, were those prepared by Ganci et al. [10]. The seeds were first dried at 60 °C in an oven and then crushed with a grinder (BioloMix, Fort Myers, FL, USA). An 8 h extraction in a Soxhlet device at 60 °C in the presence of 300 mL of hexane was used to obtain oils from grape seeds. The compositional analysis of fatty acids, carotenoids, chlorophyll and polyphenols, detailed in the same publication, is summarized in Table S1. After oil weighing, stock solutions of WGSO and RGSO were prepared at a 1:20 (*v*/*v*) dilution in FBS to ensure the maximum dispersion and bioavailability of the oily mixture. To prepare the successive working solutions, the initial stock solutions underwent further dilution within DMEM culture media.

### 4.2. Viability Assay

An MTT assay was employed to determine the cytotoxic impact of WGSO and RGSO on HepG2, CaCo-2 or diff-CaCo-2 cells [10,64]. Essentially, cells were initially seeded in 96-well plates, allowed to adhere and proliferate for 48 h, and then exposed for 24 h to WGSO and RGSO at dilutions ranging from 1:640 to 1:40. Untreated cells were used as controls. Prior to the execution of the MTT test, the morphological appearance of the cells was inspected through optical microscopy. Following the addition of MTT (Merck, Milan, Italy) and subsequent cell lysis, the optical density of the solubilized formazan was quantified at a wavelength of 550 nm. The percentage of cell viability, relative to the control, was calculated to enable the assessment of the ID_50_ for WGSO and RGSO. This analysis was performed using the ED50 PLUS V1.0 software, available online at https://www.sciencegateway.org/protocols/cellbio/drug/data/ed50v10.xls (accessed on 6 March 2025).

### 4.3. Flow Cytometry

Flow cytometric analyses, conducted as outlined in [65,66], utilized a FACSCanto instrument (BD Biosciences, Franklin Lakes, NJ, USA) for the quantification of 10,000 events. The resultant fcs files were subsequently processed using the online Floreada tool available at https://floreada.io (accessed on 20 September 2025).

The effects of treatments on cell size, internal complexity and overall integrity were preliminarily evaluated by analyzing the FSC-A and SSC-A parameters of the collected cell populations.

To determine cell cycle distribution, control and treated cells were harvested and fixed for 12 h in ice-cold 70% ethanol, followed by a PBS wash and incubation with 40 μg RNase A (Sigma)/mL and 20 μg PI (Sigma)/mL for 30 min at 37 °C prior to flow cytometric analysis.

The extent of phosphatidylserine externalization, a marker of early apoptosis, in control and treated cell samples was determined utilizing the AnnexinV-FITC Apoptosis Staining/Detection Kit (ab 14085, Abcam, Cambridge, UK). Harvested control and treated cells were incubated with 5 mL each of Annexin V-FITC II and 50 μg PI/mL for 5 min in the dark prior to flow cytometric analysis.

ROS production was quantified in accordance with the manufacturer’s protocol employing the CheKine™ Reactive Oxygen Species (ROS) Detection Fluorometric Assay Kit (Abbkine, Atlanta, GA, USA). Harvested control and treated cells were incubated with a 10 mM solution of the ROS indicator 2′,7′-dichlorodihydrofluorescein diacetate (DCFH-DA) in FBS-free DMEM for 30 min at 37 °C in the dark preceding flow cytometry analysis.

Flow cytometric evaluation of intracellular AVO accumulation was performed on collected control and treated cells that had been incubated with 1 mg acridine orange (Sigma)/mL for 15 min at room temperature.

### 4.4. Protein Blot

As already reported in [10,64], total protein samples were isolated from control and treated cells using a lysis buffer containing 7 M Urea, 2% 3-((3-cholamidopropyl) dimethylammonium)-1-propanesulfonate (CHAPS), and 10 mM dithiothreitol (DTT), all sourced from Sigma, supplemented with a cocktail of protease inhibitors (Promega, Madison, WI, USA) and quantified using the Bradford method.

For Western blot analysis, 25 mg of the samples underwent electrophoretic separation on a 13% sodium dodecyl sulphate-polyacrylamide gel electrophoresis (SDS-PAGE) system (Bio-Rad, Hercules, CA, USA), followed by their transfer onto nitrocellulose membranes. These membranes were then incubated overnight at 4°C with the following rabbit primary antibodies: anti-Bax (Y080089, ABM, Richmond, BC, Canada; working dilution 1:375), anti-Bcl-2 (TX100064, GeneTex, Irvine, CA, USA; working dilution 1:1750), anti-Beclin-1 (SAB570102, Sigma; working dilution 1:1000), anti-caspase-1 (CPA5426, Cohesion Biosciences, London, UK; working dilution 1:750), anti-caspase-3 (9662, Cell Signaling Technology, Danvers, MA, USA; working dilution 1:1000), anti-GSDMD-NT (CQA6563, Cohesion Biosciences; working dilution 1:750), anti-hsp60 (Y058412, ABM; working dilution 1:1000), anti-hsp90 (CPA9400, Cohesion Biosciences; working dilution 1:1500), anti-LC3 (L8918, Sigma; working dilution 1:750), anti-phospho-MLKL (Ser373, Tyr376) (PMLKL-140AP, FabGennix, Frisco, TX, USA; working dilution 1:750), anti-SQSTM1/p62 (SAB5700054, Sigma; working dilution 1:1000), and, as an internal control, anti-β-actin (NB-22-1460, Neo Biotech, Nanterre, France; working dilution 1:1000). For dot blot analysis, conditioned media derived from both control and treated cell cultures were harvested, concentrated with Vivaspin 500 10 kDa columns (GE-28-9322-25, Cytiva, Uppsala, Sweden) and their protein concentrations quantified by the Bradford method. Once spotted onto the nitrocellulose membrane, rabbit anti-HMGB1 antibody (PA5119191, Life Technologies, Carlsbad, CA, USA; working dilution 1:500) was applied. Following incubation of either Western or dot blot membranes with the peroxidase-conjugated secondary antibody (Ab6721, Abcam, RRID: AB_955447; working dilution 1:3000) at room temperature for 1 h, immunodetection was carried out using an enhanced chemiluminescence system (Versadoc MP Imaging System, Bio-Rad) with the SuperSignal West Pico Plus substrate (ThermoFisher, Waltham, MA, USA). Images of the whole immunoblots are shown in Figure S2. The quantification of each signal’s intensity was performed using ImageJ v.1.52a software, followed by normalization relative to the intensity of the actin band in Western blot experiments.

### 4.5. Statistics

Results are displayed as mean ± standard error of the mean (s.e.m.) of triplicate experiments. Statistical procedures involved one-way analysis of variance (ANOVA) with the Holm–Sidak post hoc test and the Shapiro–Wilk normality test carried out using the SigmaPlot 11.0 software (SYSTAT, San Jose, CA, USA).

## 5. Conclusions

Evidence has been presented regarding the cytotoxicity of seed oils from Sicilian grapes in liver and colorectal cancer cell model systems, identifying damage-promoting aspects that involve the modulation of autophagy, cell redox status and inflammatory pathways. The obtained findings highlight that variations in oils’ chemical profiles result in significantly divergent biological outcomes. They also suggest that HepG2 cells are particularly susceptible to specific components present in WGSO but absent or less abundant in RGSO, whereas intestinal epithelial cancer cells possess greater resilience to the metabolic perturbations induced by WGSO. Such differential sensitivity may stem from the metabolic specialization of hepatocytes, particularly their reliance on lipid metabolism and nutrient-sensing signaling pathways [67]. The suppression of autophagy markers observed in WGSO-treated HepG2 cells and RGSO-treated CaCo-2 cells is consistent with a functional imbalance in the upstream control systems of the autophagic process, notably the Phosphoinositide 3-Kinase (PI3K)–AKT–mechanistic Target of Rapamycin (mTOR) axis and the Uncoordinated 51-like kinase 1 (ULK1)–Beclin-1 complex [68], which deserves future exploration.

A potential limitation of this study is the asymmetrical progression of the mechanistic investigations across the two cellular systems. While the CaCo-2 model allowed for a comparative analysis of both WGSO and RGSO, the studies in HepG2 cells were focused exclusively on WGSO. This decision was dictated by the initial screening results, as the ID_50_ value for RGSO proved unattainable in the hepatic cell model within the concentration range tested. While this approach guarantees that mechanistic insights are grounded in robust dose–response data, it precludes a comparative analysis of the two oils across both cytotypes. Future research might explore different exposure windows or higher concentrations to determine if the lack of symmetry is due to cell-specific resistance or different metabolic pathways in hepatic versus intestinal environments.

Although in vitro results reveal significant biological potential, the in vivo efficacy of WGSO and RGSO hinges on factors such as absorption, distribution, metabolism, stability and delivery of their bioactive elements. Natural endogenous constituents within grape seed oils, including unsaturated fatty acids, tocopherols, phytosterols and minor phenolic fractions, are susceptible to oxidative instability during storage, digestive breakdown or suboptimal intestinal transport, which collectively reduce their systemic bioavailability [69,70]. For this reason, formulation strategies are of paramount importance. Various encapsulation techniques, such as microencapsulation, nanoemulsions, liposomal delivery systems, and polymeric nanocapsules, have proven effective for improving oxidative stability, preserving sensitive bioactives, boosting solubility, facilitating intestinal absorption, and enabling controlled release [71,72,73,74]. Such technological advancement could significantly increase the practical applicability of these oils for nutraceutical or drug-based applications [75,76]. We thus recognize that the encouraging biological outcomes detailed in this research should be complemented by supplementary investigation into bioaccessibility, pharmacokinetics, and the optimization of delivery systems [77,78].

In conclusion, given the current findings, there is a compelling rationale for further research into Sicilian WGSO and RGSO aimed at the development of innovative preventive and therapeutic options for liver and colorectal cancers, alongside their utilization as supplements in bioactive food possessing antitumor efficacy.

## Figures and Tables

**Table 1:** Biological activity of selected polyphenols contained in WGSO and RGSO. LOQ = limit of quantification, LOD = limit of detection.

Molecule	Oil/Amount (ng/g) [10]	Activity (Cytotype)	Reference
Hydroxytyrosol	WGSO/<LOQ	Down-regulation of proliferation (HepG2 cells).	[41]
	RGSO/<LOQ		
Coumaric acid	WGSO/<LOQ RGSO/39.5	Down-regulation of proliferation, increase in sub-G_0_G_1_ fraction and ROS production (colon cancer cells).	[42]
Ferulic acid	WGSO/24.0	Increase in ROS production (HepG2 cells);	[43]
	RGSO/28.0	Down-regulation of adhesion and proliferation (colon cancer cells).	[44]
Oleocanthal	WGSO/20.1 RGSO/<LOD	Decrease in viability, increase in ROS production (colon and liver cancer cells including HepG2).	[45]
Syringic acid	WGSO/153.2	Down-regulation of proliferation, increase in ROS production, increase in *BAX* expression, decrease in *BCL2* expression (HepG2 cells);	[46]
	RGSO/4213.3	Inhibition of apoptosis (CaCo-2 cells).	[47]
Rutin	WGSO/<LOQ	Down-regulation of autophagy (HepG2 cells);	[48]
	RGSO/<LOQ	Decrease in Beclin-1 levels and LC3-II/LC3-I ratio, and increase in p62 levels (HepG2 cells).	[49]
Kaempferol	WGSO/163.1 RGSO/120.9	Decrease in hsp90 levels (colon cancer cells);	[50]
		Decrease in hsp60 levels (colorectal cancer cells);	[51]
		Increase in G_0_/G_1_ cell fraction (liver cancer cells).	[52]

## Data Availability

All data generated or analyzed during this study are included in this published article.

## Supplementary Materials

The following supporting information can be downloaded at https://www.mdpi.com/article/10.3390/molecules31101567/s1: Figure S1: Phase contrast micrographs of HepG2, CaCo-2 and diff-CaCo-2 cells exposed for 24 h to different dilutions of WGSO or RGSO in complete DMEM medium; Figure S2: Left: Images depicting the complete protein blots stained with Ponceau S, as well as the molecular weight markers (M) for reference (in Western blots). Right: Images depicting the total protein immunoblots; Table S1: Compositional analysis of WGSO and RGSO (from [10]).
