# Supplementary material for: Cytotoxic Activity of Sicilian Red- and White-Grape Seed Oils on Human Liver and Colorectal Cancer Cells
Source: Molecules. 2026 May 8;31(10):1567. doi: 10.3390/molecules31101567 (PMC13209378; doi:10.3390/molecules31101567)
Supplement: Supplementary file 1 [file molecules-31-01567-s001.zip › molecules-4249662-supplementary.pdf]

**Cytotoxic Activity of Sicilian Red- and White-Grape Seed Oils on Human Liver and Colorectal Cancer Cells**

Daniela Ganci , Giulia Abruscato , Roberto Chiarelli , Manuela Mauro,
Vincenzo Arizza, Mirella Vazzana and Claudio Luparello

SUPPLEMENTARY MATERIAL

**HEPG2 cells + WGSO**

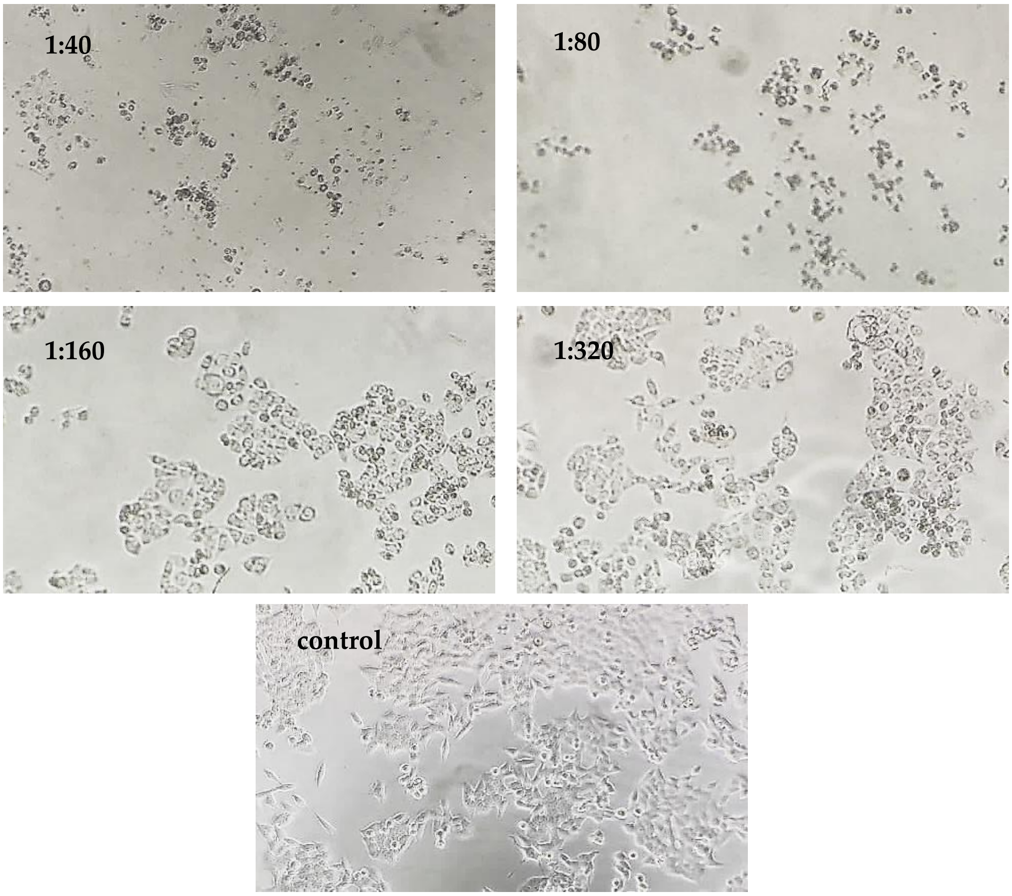


**HEPG2 cells + RGSO**

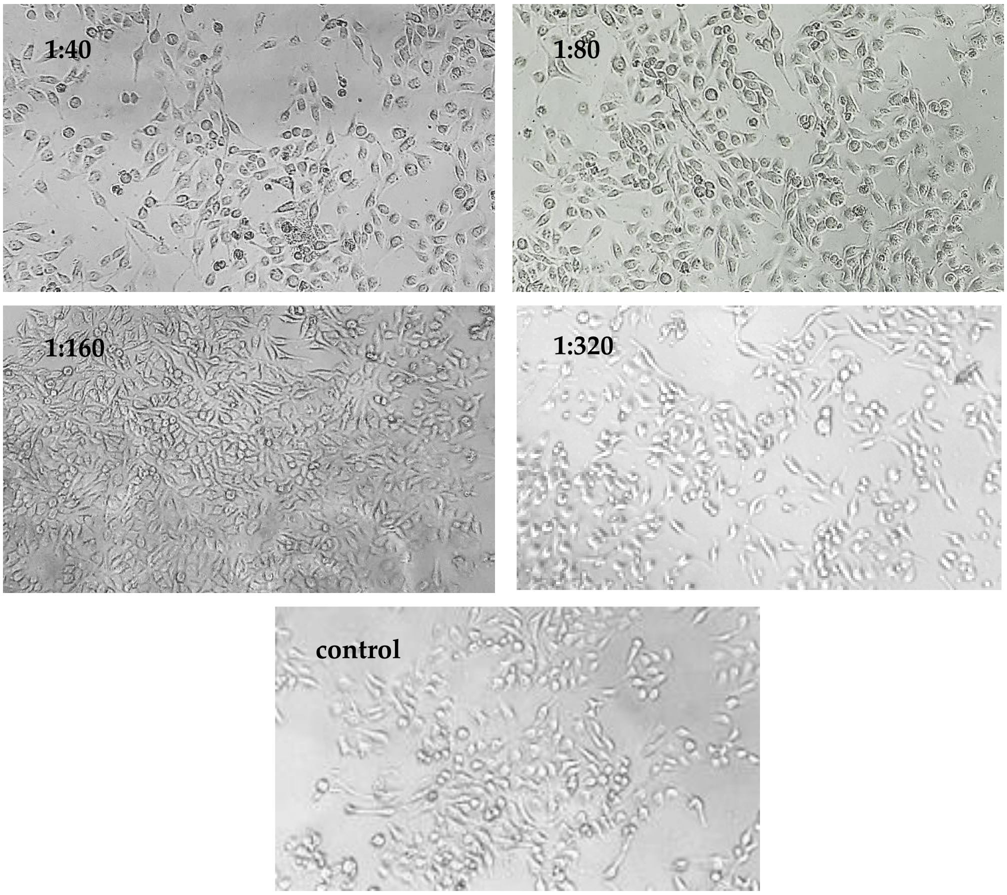

**CaCo-2 cells + WGSO**

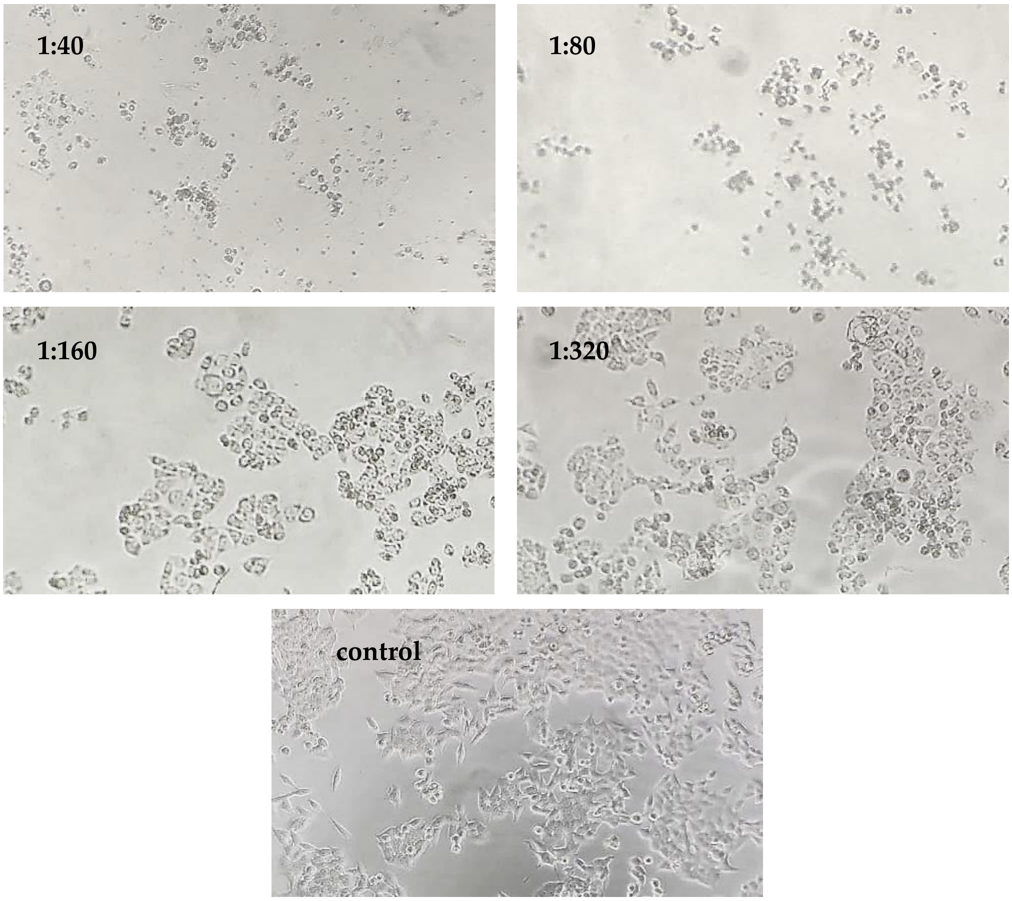


**CaCo-2 cells + RGSO**

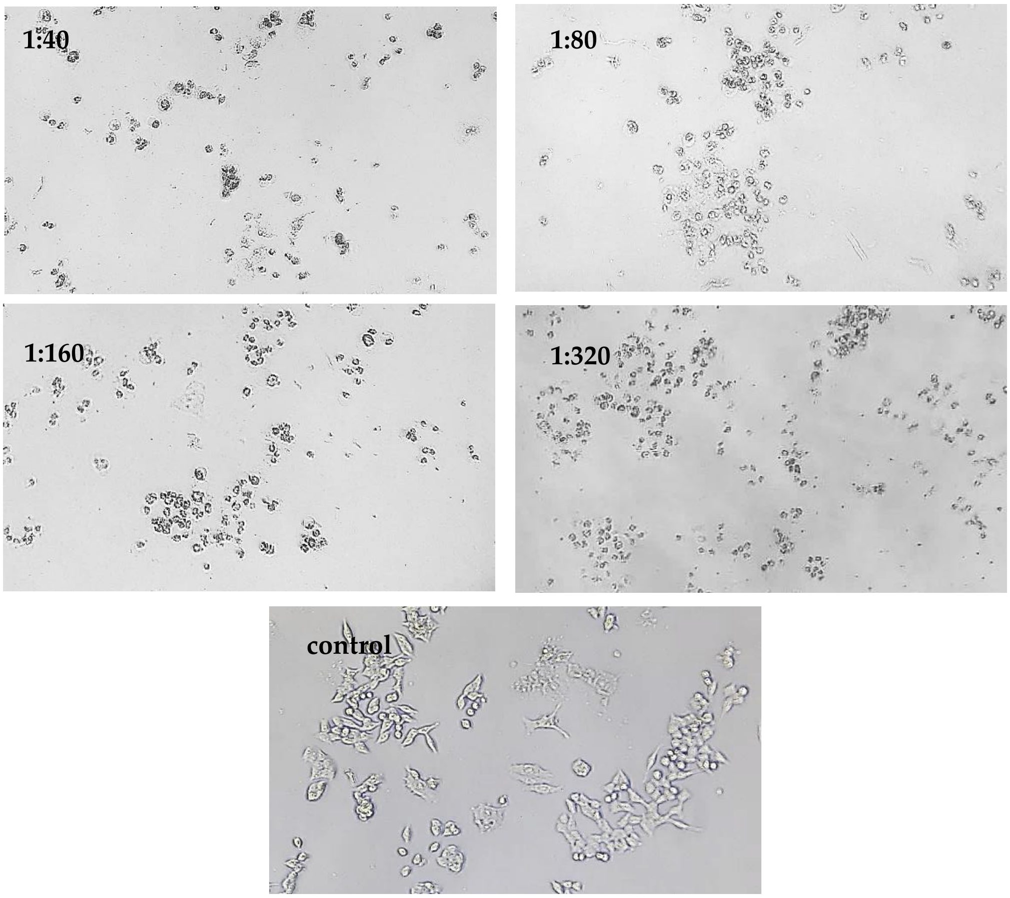

**Diff-CaCo-2 cells + WGSO**

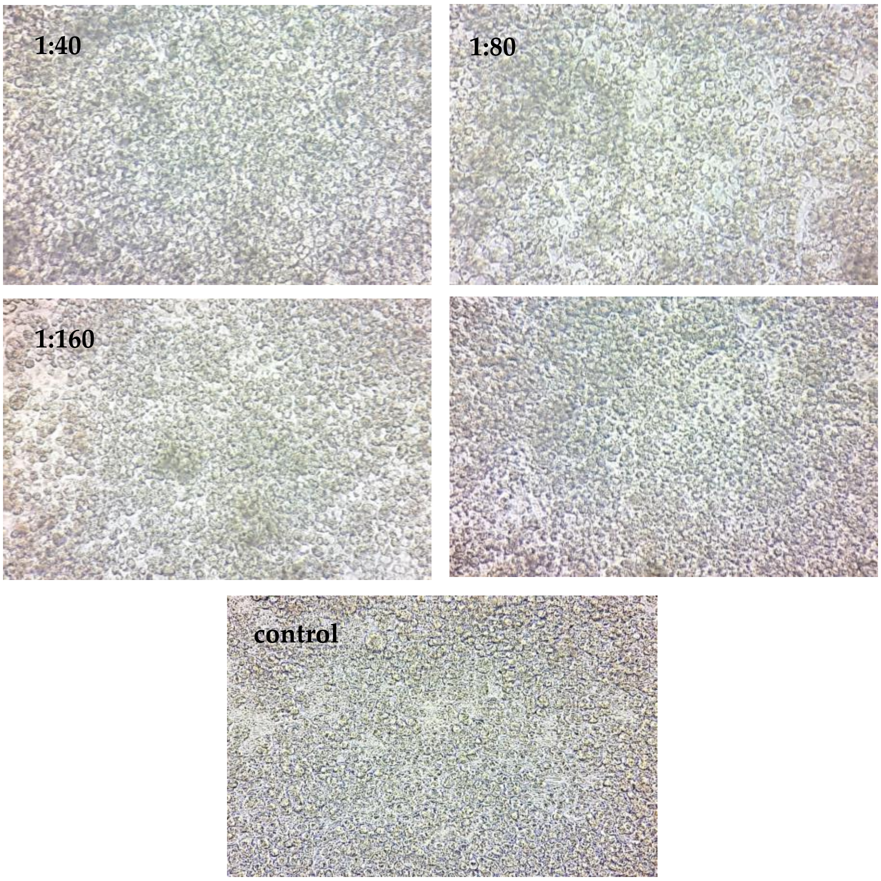


**Diff-CaCo-2 cells + RGSO**

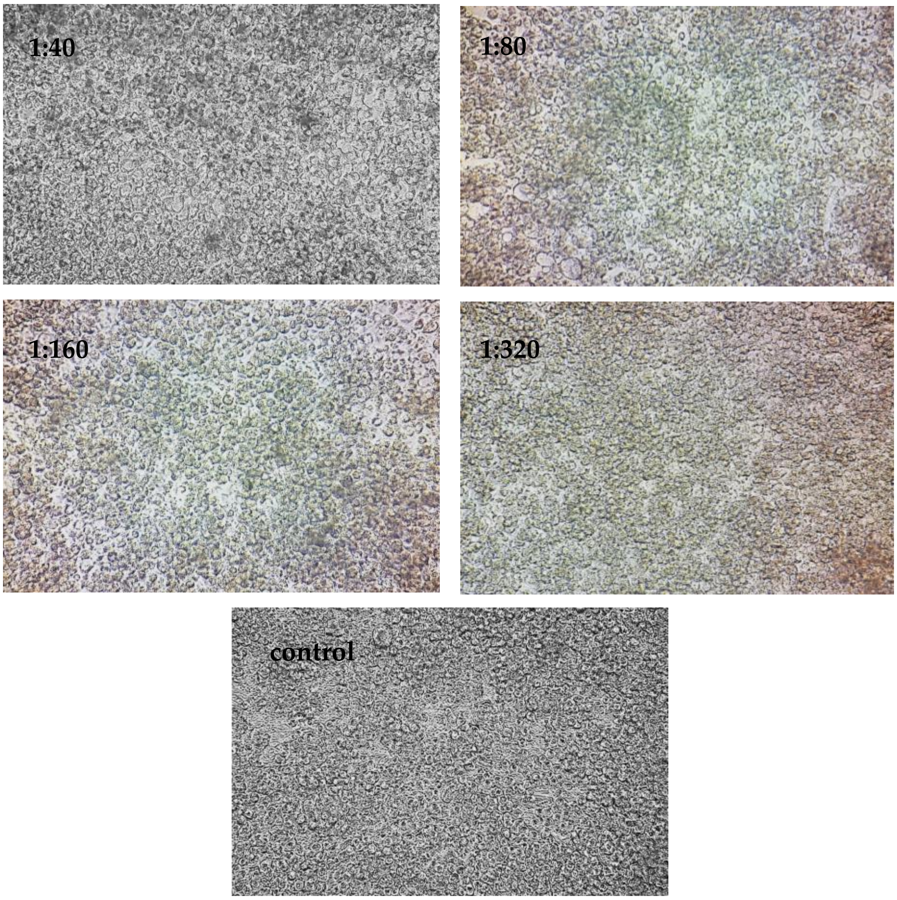


**Figure S1**: Phase contrast micrographs of HepG2, CaCo-2 and diff-CaCo-2 cells exposed for 24 h to different dilutions of WGSO or RGSO in complete DMEM medium. Microscopic magnification 20x.

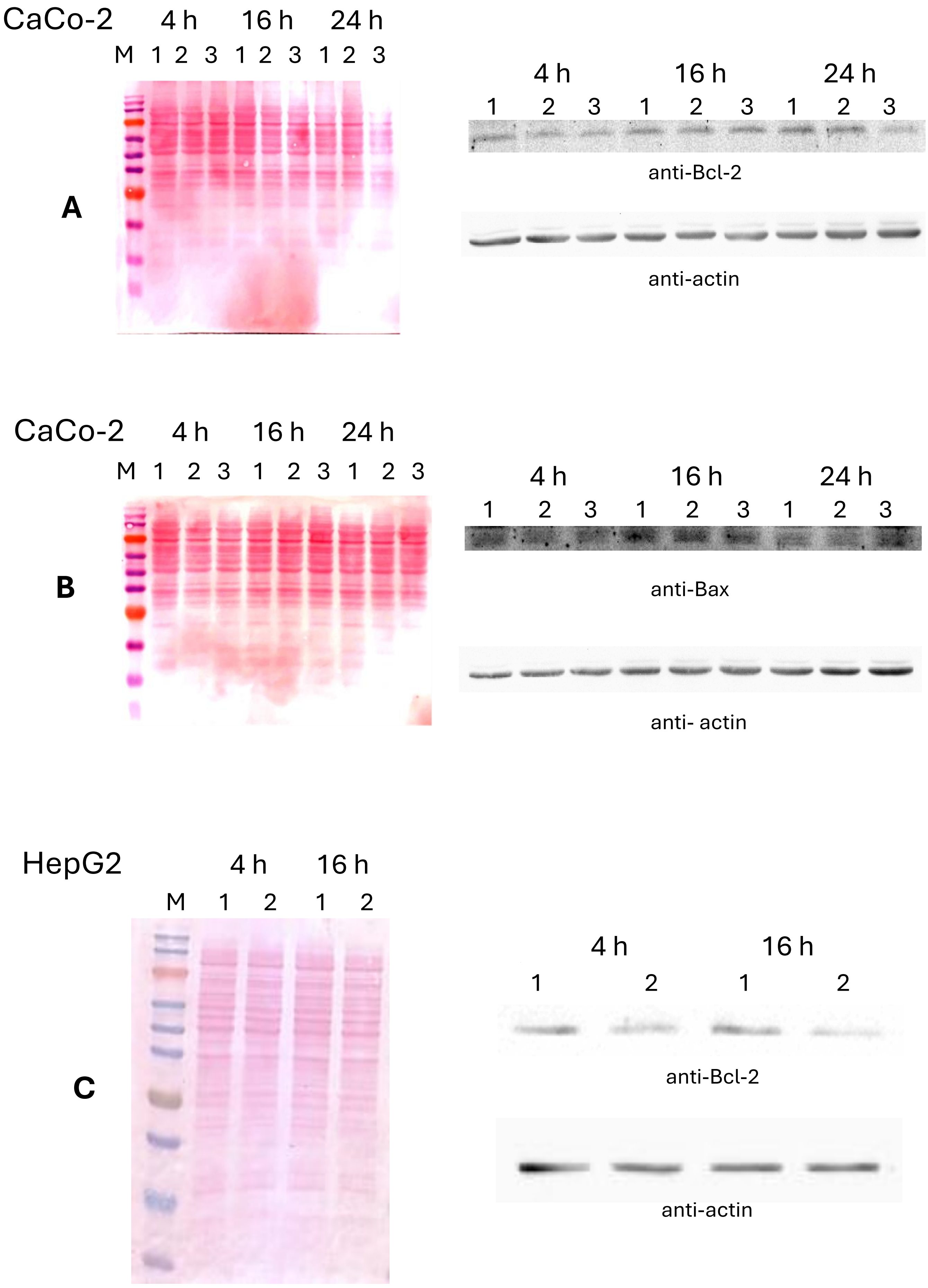
CaCo-2
4 h
16 h
24 h
M 1 2 3 1 2 3 1 2 3
A
1 2 3 1 2 3 1 2 3
anti-Bcl-2
anti-actin
B
anti-Bax
anti- actin
HepG2
M 1 2 1 2
C
1 2 1 2
anti-Bcl-2
anti-actin

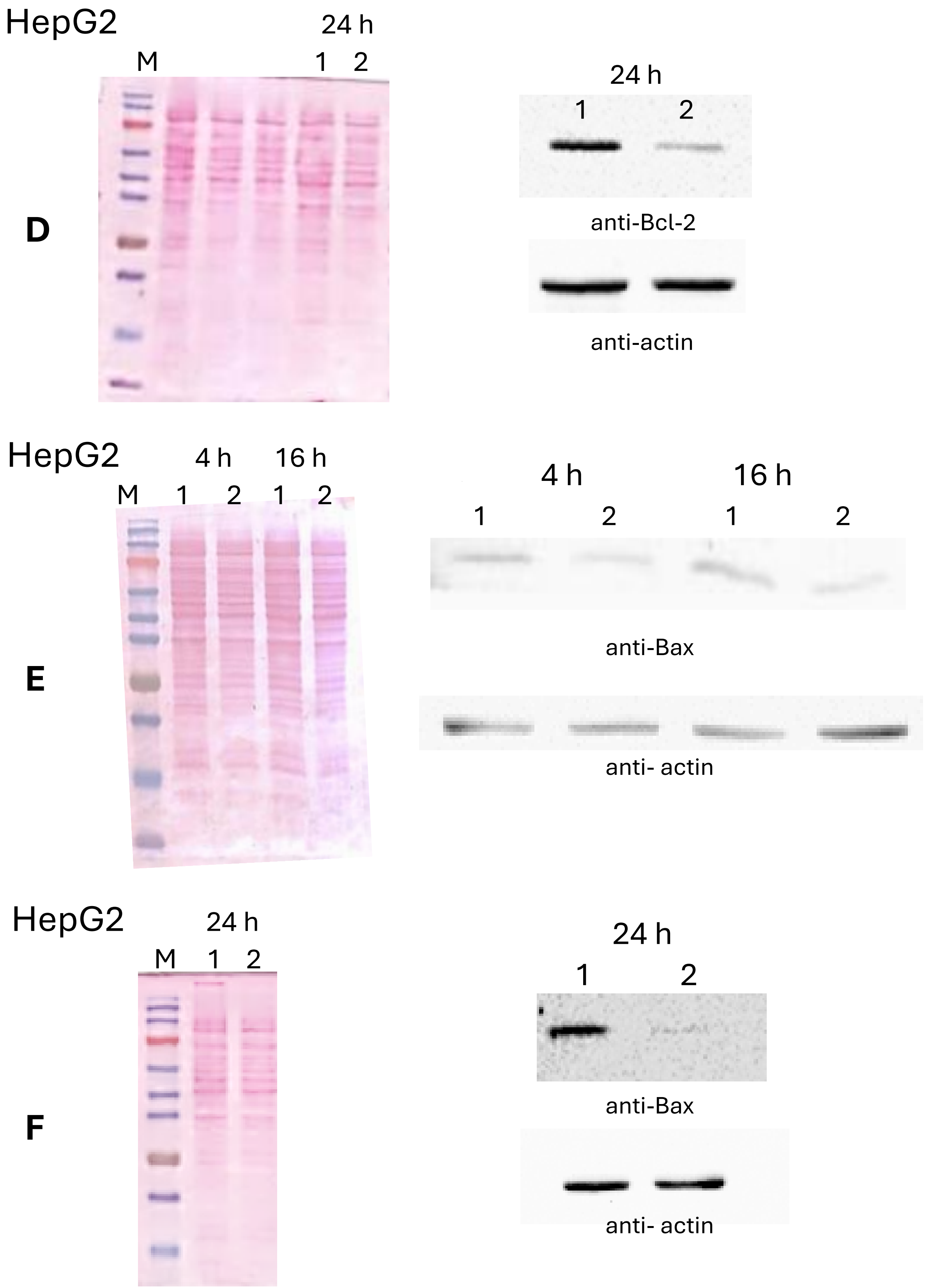
HepG2
24 h
M
1 2
D
24 h
1 2
anti-Bcl-2
anti-actin
HepG2
4 h 16 h
M 1 2 1 2
E
4 h 16 h
1 2 1 2
anti-Bax
anti- actin
HepG2
24 h
M 1 2
F
24 h
1 2
anti-Bax
anti- actin

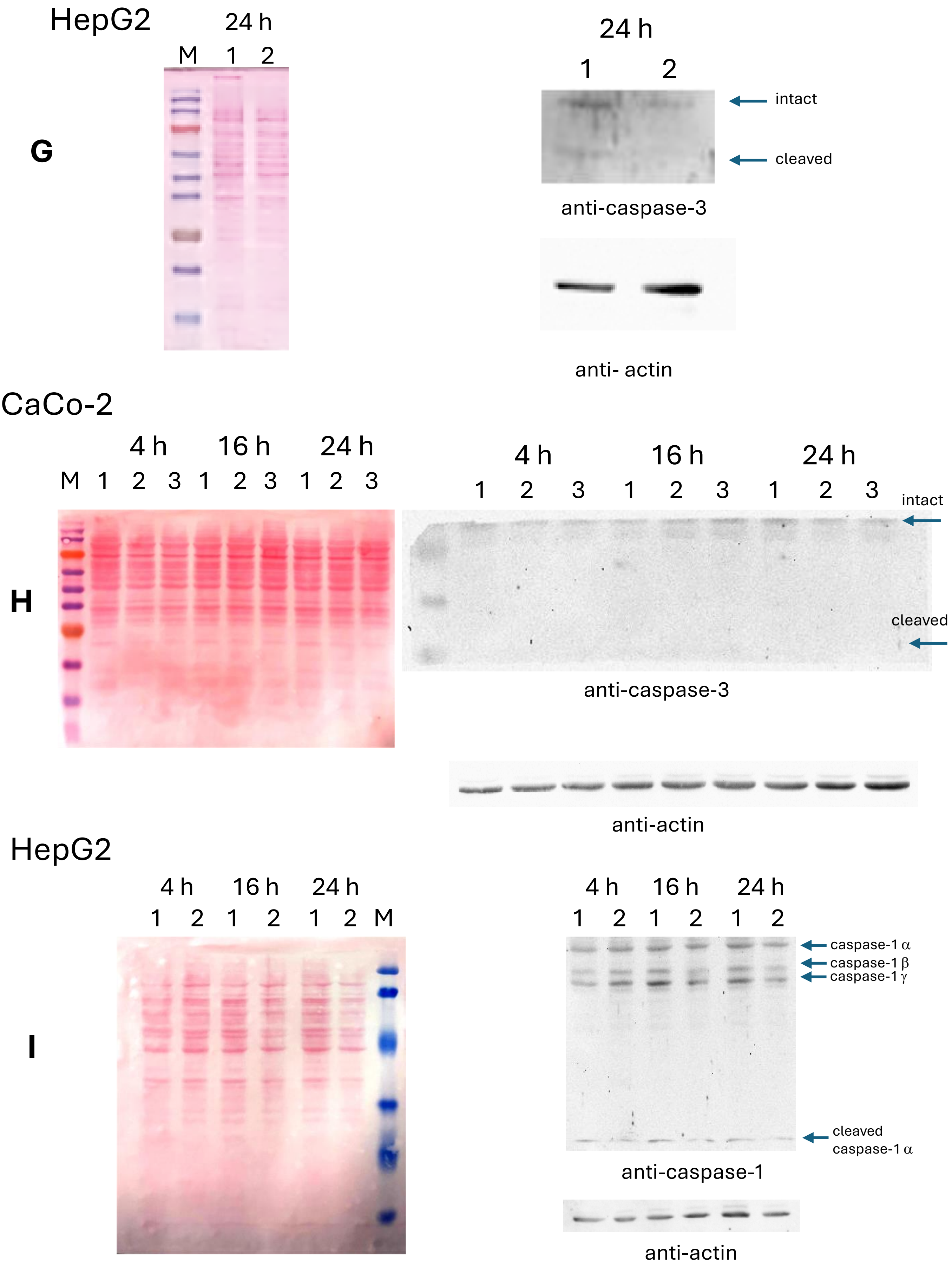

HepG2
24 h
M 1 2
G
24 h
1 2
intact
cleaved
anti-caspase-3
anti- actin
CaCo-2
4 h 16 h 24 h
M 1 2 3 1 2 3 1 2 3
H
4 h 16 h 24 h
1 2 3 1 2 3 1 2 3
intact
cleaved
anti-caspase-3
anti-actin
HepG2
4 h 16 h 24 h
1 2 1 2 1 2 M
I
4 h 16 h 24 h
1 2 1 2 1 2
caspase-1 α
caspase-1 β
caspase-1 γ
cleaved caspase-1 α
anti-caspase-1
anti-actin

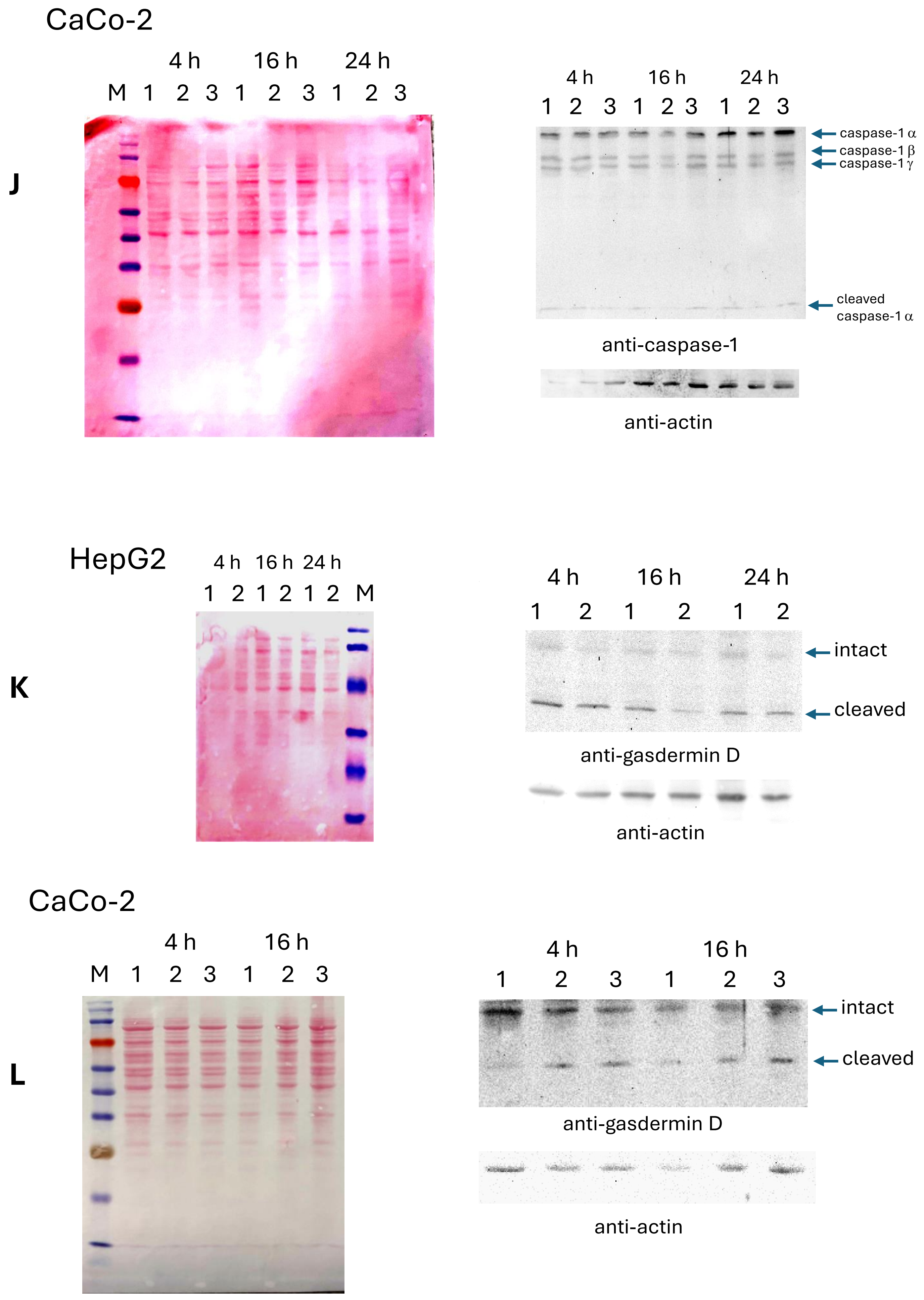
J
CaCo-2
4 h 16 h 24 h
M 1 2 3 1 2 3 1 2 3
4 h 16 h 24 h
1 2 3 1 2 3 1 2 3
caspase-1 α
caspase-1 β
caspase-1 γ
cleaved caspase-1 α
anti-caspase-1
anti-actin
K
HepG2
4 h 16 h 24 h
1 2 1 2 1 2 M
4 h 16 h 24 h
1 2 1 2 1 2
intact
cleaved
anti-gasdermin D
anti-actin
L
CaCo-2
4 h 16 h
M 1 2 3 1 2 3
4 h 16 h
1 2 3 1 2 3
intact
cleaved
anti-gasdermin D
anti-actin

**M**

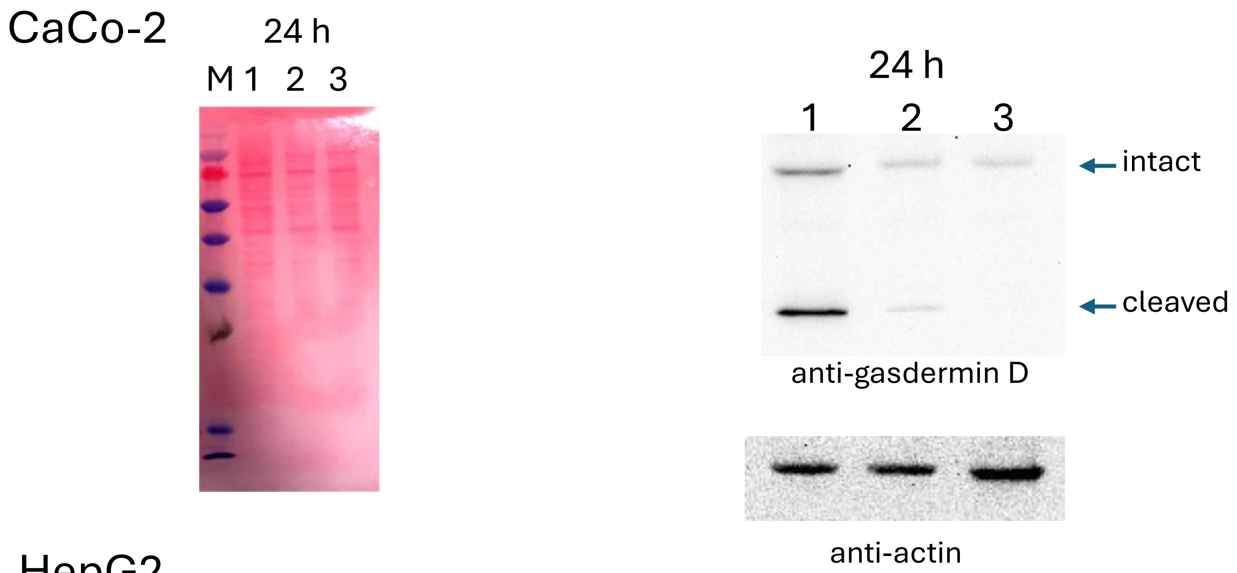
CaCo-2
24 h
M 1 2 3
24 h
1 2 3
intact
cleaved
anti-gasdermin D
anti-actin

**N**

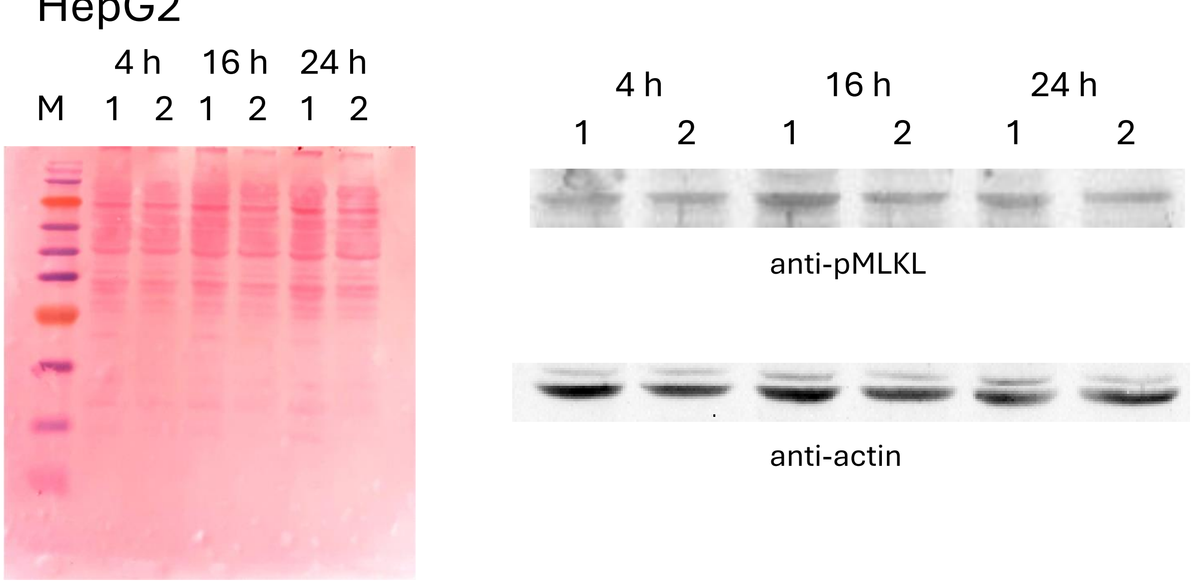
HepG2
4 h 16 h 24 h
M 1 2 1 2 1 2
4 h 16 h 24 h
1 2 1 2 1 2
anti-pMLKL
anti-actin

**O**

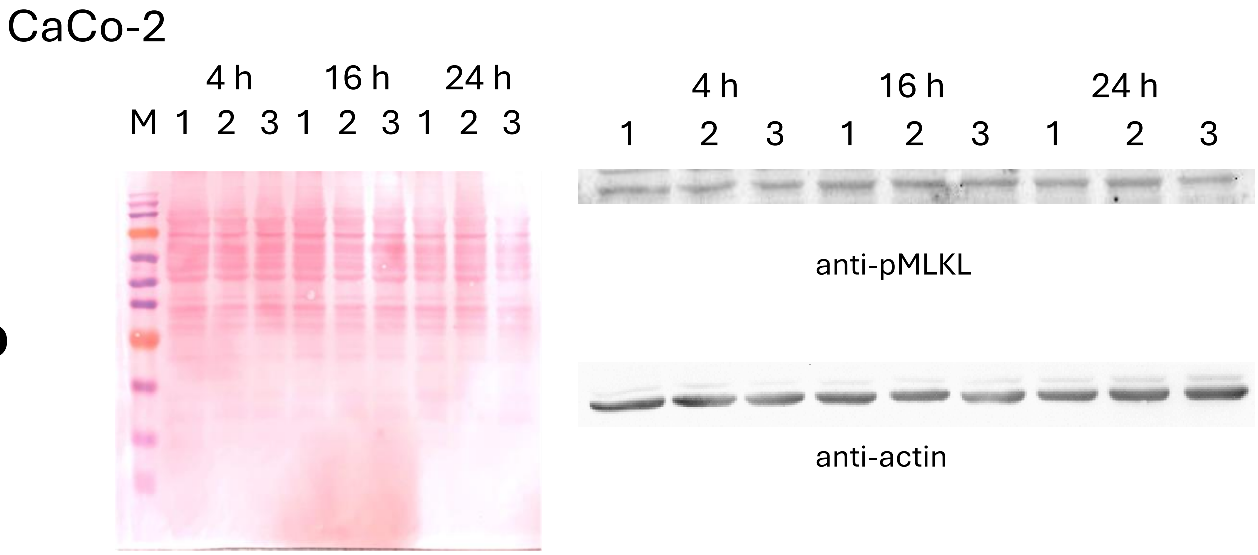
CaCo-2
4 h 16 h 24 h
M 1 2 3 1 2 3 1 2 3
4 h 16 h 24 h
1 2 3 1 2 3 1 2 3
anti-pMLKL
anti-actin

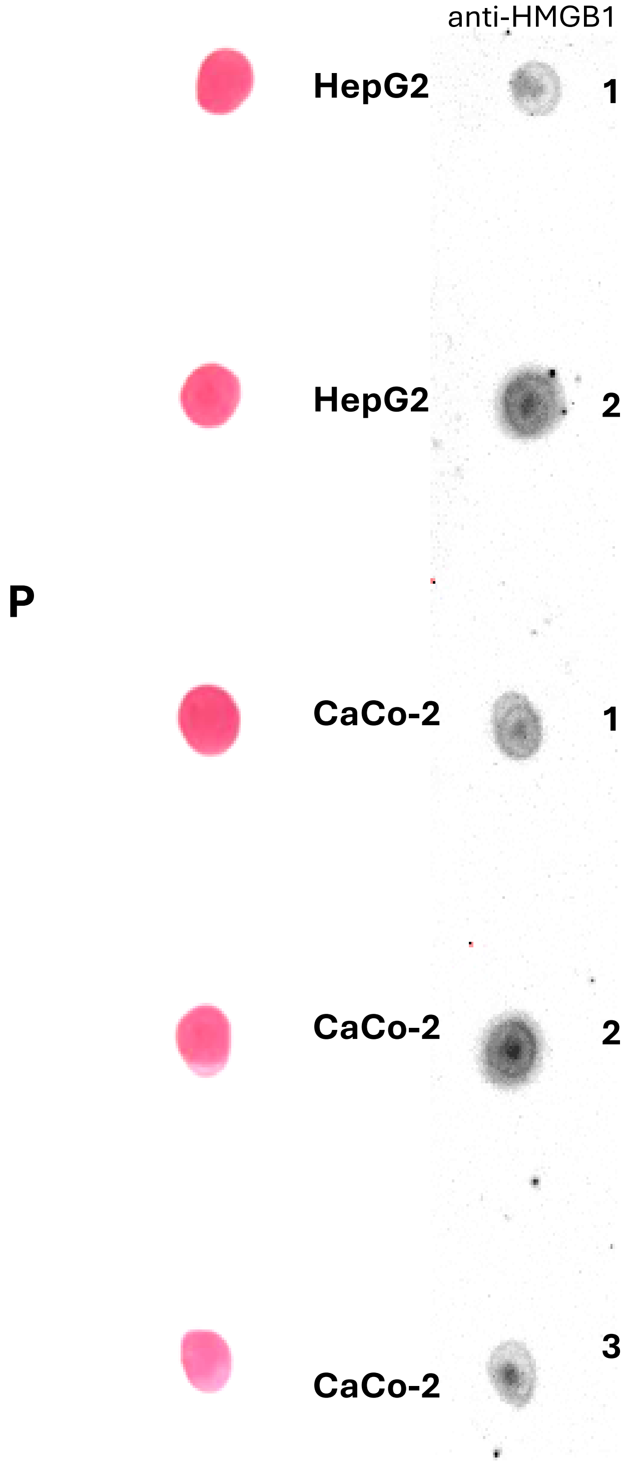

anti-HMGB1
HepG2
1
HepG2
2
P
CaCo-2
1
CaCo-2
2
3
CaCo-2

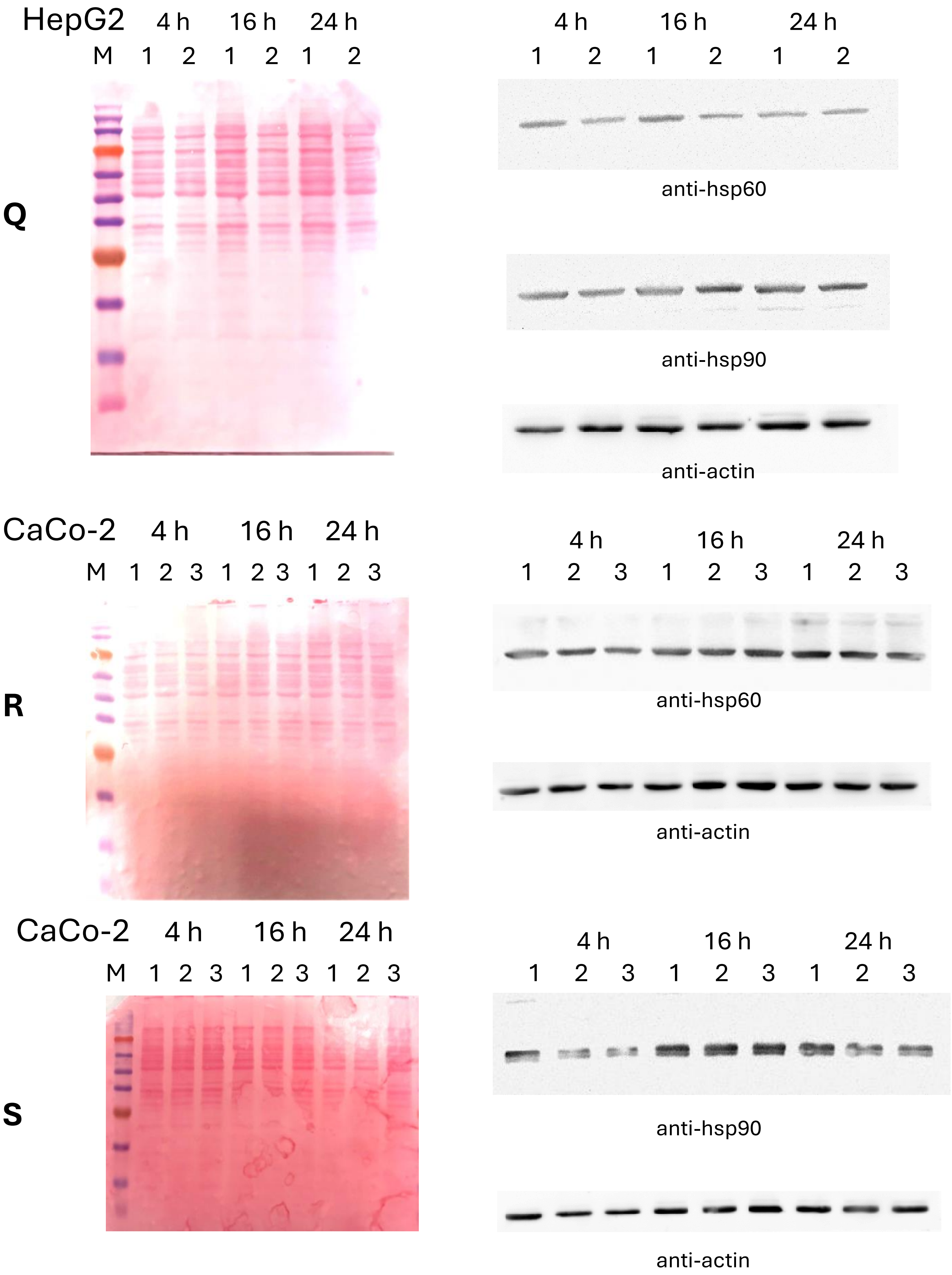
HepG2 4 h 16 h 24 h
M 1 2 1 2 1 2
Q
4 h 16 h 24 h
1 2 1 2 1 2
anti-hsp60
anti-hsp90
anti-actin
CaCo-2 4 h 16 h 24 h
M 1 2 3 1 2 3 1 2 3
R
4 h 16 h 24 h
1 2 3 1 2 3 1 2 3
anti-hsp60
anti-actin
CaCo-2 4 h 16 h 24 h
M 1 2 3 1 2 3 1 2 3
S
4 h 16 h 24 h
1 2 3 1 2 3 1 2 3
anti-hsp90
anti-actin

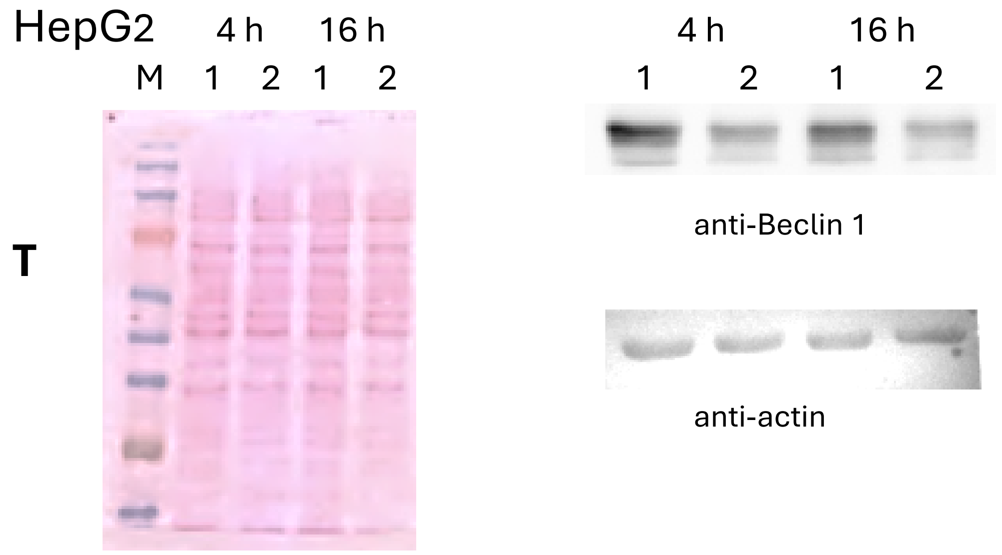
HepG2
4 h
16 h
M 1 2 1 2
T
anti-Beclin 1
anti-actin

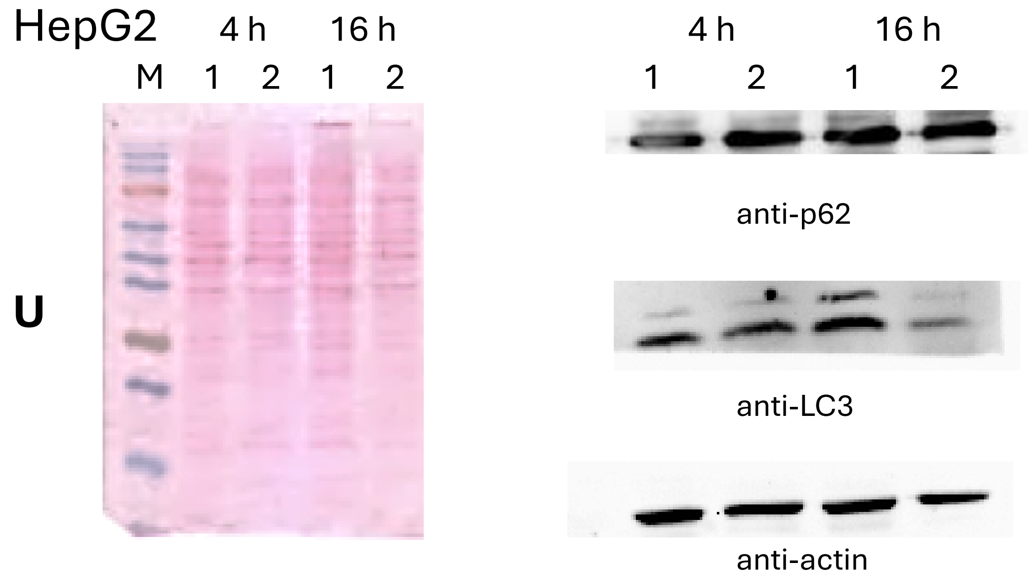
HepG2
4 h
16 h
M 1 2 1 2
U
anti-p62
anti-LC3
anti-actin

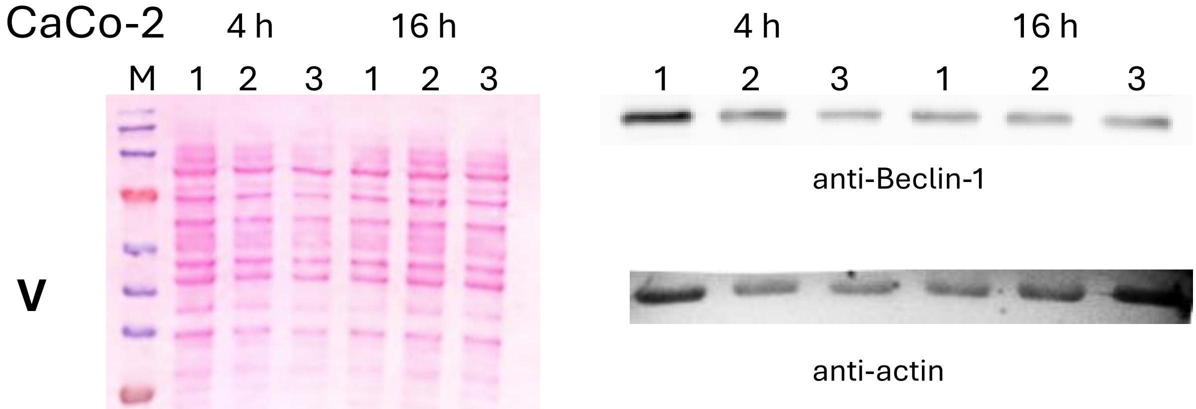
CaCo-2
4 h
16 h
M 1 2 3 1 2 3
V
anti-Beclin-1
anti-actin

W

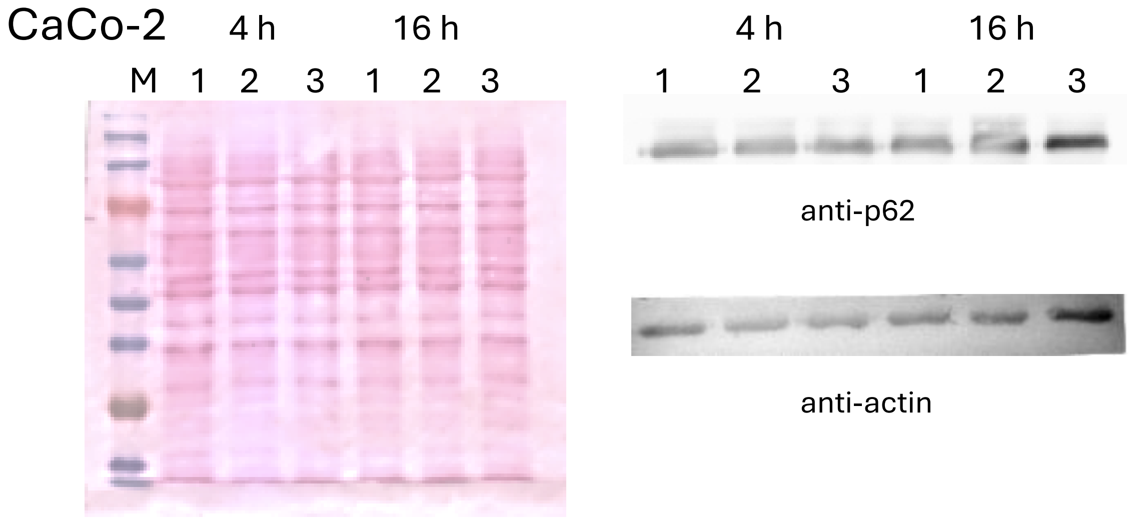


X

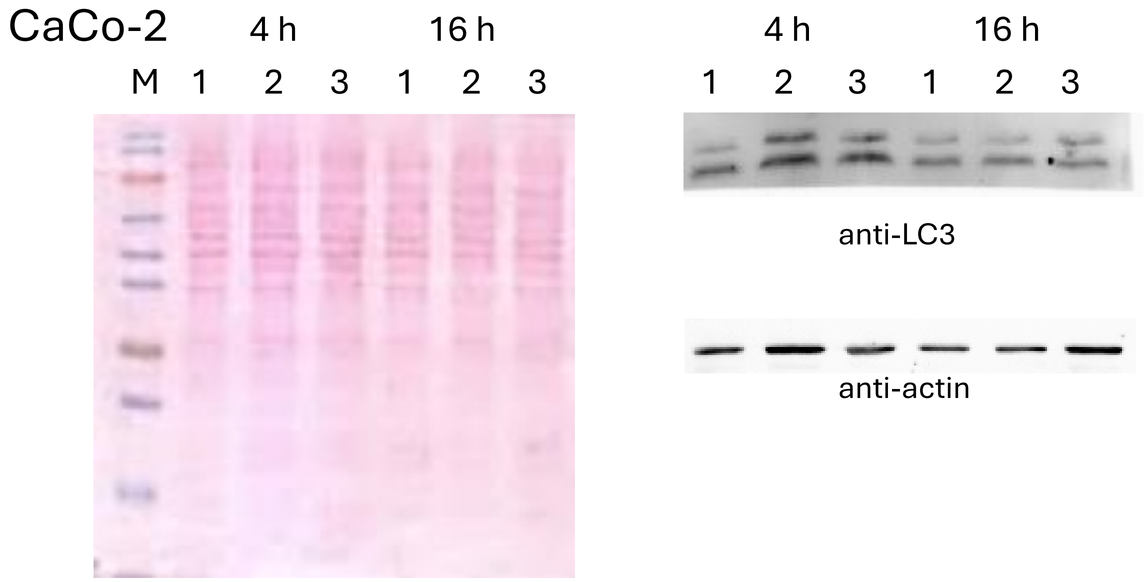


**Figure S2**: Left: Images depicting the complete protein blots stained with Ponceau S, as well as the molecular weight markers (M) for reference (in Western blots). Right: Images depicting the total protein immunoblots. Control = sample 1, WGSO = sample 2, RGSO = sample 3

**Table S1. Compositional analysis of WGSO and RGSO (from [10])**

| **Fatty acids (%)** | WGSO | RGSO |
|---|---|---|
| C14:0 | 0.1 | 0.1 |
| C16:0 | 8.0 | 7.8 |
| C16:1 ω7 cis | 0.04 | 0.03 |
| C17:0 | 0.1 | 0.05 |
| C18:0 | 4.1 | 4.2 |
| C18:1 ω9 cis | 19.9 | 16.8 |
| C18:1 ω7 cis | 1.2 | 1.2 |
| C18:2 ω6,9 cis, cis | 65.7 | 68.8 |
| C20:0 | 0.1 | 0.1 |
| C18:3 ω3 + C20:1 ω9 | 0.5 | 0.5 |
| C20:2 | not determined | 0.03 |
| C22:0 | not determined | 0.03 |
| **Total carotenoids (mg/Kg)** | 0.02 | 0.2 |
| **Total chlorophyll (mg/Kg)** | not determined | 0.02 |
| **Polyphenols (ng/g)** | | |
| Hydroxytyrosol | <limit of quantification | <limit of quantification |
| Coumaric Acid | <limit of quantification | 39.5 |
| Ferulic Acid | 24.0 | 28.0 |
| Oleacein | 10.5 | <limit of quantification |
| Oleocanthal | 20.1 | <limit of detection |
| Syringic Acid | 153.2 | 4213.3 |
| Trans-OH-Cinnamic | 61.4 | 323.5 |
| Rutin | <limit of quantification | <limit of quantification |
| Kaempferol | 163.1 | 120.9 |
